# Supplementary material for: Impact of progressive resistance training on CT quantified muscle and adipose tissue compartments in pancreatic cancer patients
Source: PLoS One. 2020 Nov 30;15(11):e0242785. doi: 10.1371/journal.pone.0242785 (PMC7703876; doi:10.1371/journal.pone.0242785)
Supplement: S1 Table — n = 22. TFA = total fat area, VFA = visceral fat area, SFA = subcutaneous fat area, VFR = visceral fat ratio, MA = muscle area, IMFA = inter-muscular-fat area, SMI = skeletal muscle index, MD = muscle density (in HU); paired t-test; * = significant. (DOCX) [file pone.0242785.s002.docx]

S1 Table. CT quantified body compartments with a baseline CT after surgery.

|  | **T0** | **T2** | **Difference** | **p-value** |
| --- | --- | --- | --- | --- |
| TFA (cm²) | 285.9 | 268.7 | -17.2 | 0.299 |
| VFA (cm²) | 116.4 | 102.0 | -14.4 | 0.077 |
| SFA (cm²) | 158.6 | 156.3 | -2.3 | 0.794 |
| IFA (cm²) | 11.0 | 10.4 | -0.6 | 0.384 |
| VFR | 0.75 | 0.74 | -0.1 | 0.859 |
| MA_150_ (cm²) | 141.0 | 145.5 | 4.5 | 0.031* |
| MD_150_ (HU) | 44.0 | 47.5 | 3.5 | 0.007* |
| SMI_150_ (cm²/m²) | 45.7 | 47.1 | 1.4 | 0.030* |
| MA_100_ (cm²) | 90.3 | 104.2 | 13.9 | 0.001* |
| MD_100_ (HU) | 57.6 | 59.1 | 1.5 | 0.021* |
| SMI_100_ (cm²/m²) | 29.3 | 33.6 | 4.3 | 0.001* |

N= 22. TFA= total fat area, VFA= visceral fat area, SFA= subcutaneous fat area, VFR= visceral fat ratio, MA= muscle area, IMFA= inter-muscular-fat area, SMI= skeletal muscle index, MD= muscle density (in HU); paired t-test; *= significant
