# Supplementary material for: Impact of progressive resistance training on CT quantified muscle and adipose tissue compartments in pancreatic cancer patients
Source: PLoS One. 2020 Nov 30;15(11):e0242785. doi: 10.1371/journal.pone.0242785 (PMC7703876; doi:10.1371/journal.pone.0242785)
Supplement: S2 Table — n = 6. TFA = total fat area, VFA = visceral fat area, SFA = subcutaneous fat area, VFR = visceral fat ratio, MA = muscle area, IMFA = inter-muscular-fat area, SMI = skeletal muscle index, MD = muscle density (in HU); paired t-test; * = significant. (DOCX) [file pone.0242785.s003.docx]

S2 Table. CT quantified body compartments with a Baseline CT before surgery.

|  | **T0** | **T2** | **Difference** | **p-value** |
| --- | --- | --- | --- | --- |
| TFA (cm²) | 514.9 | 295.1 | -219.8 | 0.013* |
| VFA (cm²) | 261.3 | 135.4 | -125.9 | 0.011* |
| SFA (cm²) | 235.1 | 149.3 | -85.8 | 0.045* |
| IFA (cm²) | 18.4 | 10.5 | -7.9 | 0.005* |
| VFR | 1.19 | 1.05 | -0.14 | 0.503 |
| MA_150_ (cm²) | 156.7 | 149.5 | -7.2 | 0.393 |
| MD_150_ (HU) | 44.9 | 44.8 | -0.1 | 0.981 |
| SMI_150_ (cm²/m²) | 50.2 | 48.0 | -2.2 | 0.423 |
| MA_100_ (cm²) | 102.0 | 97.7 | -4.3 | 0.708 |
| MD_100_ (HU) | 58.8 | 58.4 | -0.4 | 0.831 |
| SMI_100_ (cm²/m²) | 32.7 | 31.6 | -1.1 | 0.790 |

N= 6. TFA= total fat area, VFA= visceral fat area, SFA= subcutaneous fat area, VFR= visceral fat ratio, MA= muscle area, IMFA= inter-muscular-fat area, SMI= skeletal muscle index, MD= muscle density (in HU); paired t-test; *= significant
