# Supplementary material for: Impact of progressive resistance training on CT quantified muscle and adipose tissue compartments in pancreatic cancer patients
Source: PLoS One. 2020 Nov 30;15(11):e0242785. doi: 10.1371/journal.pone.0242785 (PMC7703876; doi:10.1371/journal.pone.0242785)
Supplement: S1 File — (PDF) [file pone.0242785.s004.pdf]

Study Protocol

SUPPORT – Study

Supervised Progressive Resistance Training for Pancreatic Cancer Patients: a randomized controlled intervention trial

Trial Registration
The trial is registered at ClinicalTrials.gov as NCT.

Funding:
The study has been supported by Deutsche Krebshilfe e.V. (German Cancer Aid); Grant No. 110513, Grant received April 26, 2013

Deutsche Krebshilfe
HELFEN. FORSCHEN. INFORMIEREN.

Table of contents

Roles and Responsibilities 3
Summary 5
1. Background 6
1.1 Clinical and epidemiological background on pancreatic cancer 6
1.2 Randomized controlled trial(s) of exercise among pancreatic cancer patients 6
1.3 Own preliminary work 7
2. Study Design 8
2.1 Goals of the study / Expected Results 8
2.2 Objectives 8
2.3 Study type / setting 9
2.4 Outcomes 9
2.5 Sample size calculation 10
2.6 Inclusion and exclusion criteria 10
2.7 Randomization procedure 11
3. Interventions 11
4. Plan of investigation 13
4.1 Screening and recruitment 13
4.2 Baseline assessment 14
4.3 Data collection procedures 14
4.4 Data collection methods 15
4.4.1 Questionnaires 15
4.4.2 Exercise performance tests and physical function 16
4.4.3 Further assessments & documentation 17
4.4.4 Lab methods 18
4.5 Data management and quality control 18
4.6 Statistical analyses 18
5. Potential unexpected side-effects for study participants 19
5.1 Interventions 19
5.2 Diagnostic procedures 19
5.2.1 Questionnaires 19
5.2.2 Exercise performance tests and physical function 20
5.3 Contraindications and abortion criteria 20
6. Legal and ethical aspects 20
6.1 Ethical committee and data privacy 20
6.2 Informed Consent 21
6.3 Data storage & publication intentions 21
6.4 Insurance 21

7. References 22
8. Attachments 26

Roles and Responsibilities

Principal Investigators (PIs)

Prof. Dr. rer. nat. Karen Steindorf (Coordinator)
Head, Unit of Physical Activity and Cancer
Division of Preventive Oncology (G110)
National Center for Tumor Diseases (NCT)
German Cancer Research Center (DKFZ)
Im Neuenheimer Feld 460
D- 69120 Heidelberg
Phone: +49-(0)6221-422351 and -565838
Fax: +49-(0)6221-42-2229
Email: k.steindorf@dkfz.de

Prof. Dr. med. Thilo Hackert
Head, Division of Pancreatic Surgery
Department of General Surgery
University of Heidelberg
Im Neuenheimer Feld 110
69120 Heidelberg
Phone: +49 6221-56-5950
Fax: +49 6221-56-5969
Email: thilo.hackert@med.uni-heidelberg.de

Dr. phil. Joachim Wiskemann
Co-Head, Unit of Physical Activity and Cancer
Divisions of Preventive Oncology and of Medical Oncology
National Center for Tumor Diseases (NCT)
German Cancer Research Center (DKFZ)
University Clinic Heidelberg
Im Neuenheimer Feld 460
69120 Heidelberg
Phone: +49 6221 56-5904
Telefax: +49 6221 56-5231
Email: joachim.wiskemann@nct-heidelberg.de

Prof. Dr. Cornelia Ulrich
Head, Division of Preventive Oncology (G110)
National Center of Tumor Diseases (NCT) Heidelberg
Im Neuenheimer Feld 460
D-69120 Heidelberg
Phone: +49-(0)6221-56 5528
Fax: +49-(0)6221-56 5231
Email: neli.ulrich@nct-heidelberg.de

Biometricians

Prof. Dr. rer. nat. Karen Steindorf
Head, Unit of Physical Activity and Cancer
Division of Preventive Oncology (G110)
National Center for Tumor Diseases (NCT)
German Cancer Research Center (DKFZ)
Im Neuenheimer Feld 460
D- 69120 Heidelberg
Phone: +49-(0)6221-422351 and -565838
Fax: +49-(0)6221-42-2229
Email: k.steindorf@dkfz.de

and

Dr. sc. hum. Thomas Bruckner
Institute of Medical Biometry and Informatics
University of Heidelberg
Im Neuenheimer Feld 305
D-69120 Heidelberg
Phone: +49-(0)6221-56-4371
Fax: +49-(0)6221-56-4195
E-Mail: bruckner@imbi.uni-heidelberg.de

Study Sponsor

Deutsches Krebsforschungszentrum (German Cancer Research Center)
Im Neuenheimer Feld 280
D- 69120 Heidelberg
Germany

Data Safety Monitoring Committee

Members are as follows and represent expertise on pancreatic cancer, internal medicine and statistics:

- Prof. Dr. Annette Kopp-Schneider (Head, Div. of Biostatistics, German Cancer Research Center, Heidelberg)
- Dr. Dr. med. Christoph Springfeld (Attending Senior Physician; Dept. of Medical Oncology, National Center for Tumor Diseases, Heidelberg)

Heidelberg, 30th July 2013

K. Steindorf

Prof. Dr. Karen Steindorf
(Study coordinator – on behalf of the team)

Summary

Pancreatic cancer is the 4th most common cause of cancer death in Germany. The median survival time of patients with surgical resection increased over the last decades to up to 30 months. Before and shortly after pancreatic resections, the quality of life is dramatically reduced in patients compared to normal controls, and quality of life only slowly improves after surgery. Thus, quality of life among pancreatic cancer patients has become a major concern. In addition to psychological distress, predominantly the physical functioning, a major component of quality of life, is reduced after a pancreatic resection for cancer. Exercise and resistance training have been found to effectively improve quality of life in patients with various cancer types. However, to date, no resistance training intervention study among pancreatic cancer patients has been reported.

Our randomized-controlled balanced-parallel-group trial in 201 adult pancreatic cancer patients (stage I-IV) from the University Surgery Clinic in Heidelberg and further cooperation clinics within the region will compare two specific exercise interventions and one standard care control arm. The intervention programs will be (1) a 6-month supervised moderate-to-high-intensity, progressive resistance training, and (2) a 6-month home-based exercise training. Thus, the two interventions differ primarily by the intensity and mode-of-administration of the intervention. Both interventions will start at the earliest 8 weeks after surgery to allow for wound healing until maximum 12 months after the surgery. The supervised progressive training will start with only moderate-intensity training during the first 4 weeks. Only patients without comorbidities that preclude participation in the intervention arms (e.g. severe pain, heart insufficiency > NYHA III, reduced standing or walking ability) are eligible.

Our primary objective is to compare the effects of the different interventions on physical functioning, measured as change in the quality of life questionnaire EORTC-QLQ-C30 from baseline to 6 months. Additional endpoints are overall quality of life from EORTC QLQ-C30 and the pancreatic cancer specific module QLQ-PAN26, fatigue, endurance (VO_{2peak}) and strength performance (isometric and isokinetic), adherence to the interventions, discontinuation of adjuvant therapy, body weight and composition, disease progression, overall and progression-free survival. All outcomes will be assessed every 3 months for a minimum follow-up period of 12 months. Study visits will be in parallel to the standard patient management in the outpatient clinic.

Assuming a maximum drop-out rate of 25% (primarily due to death), we expect 50 evaluable patients per group. The primary efficacy analysis is based on a closed testing procedure: The global between-group comparison of mean changes of all three groups will be tested within an analysis of covariance model. In case of rejection of the global null hypothesis, all pairwise comparisons will be based on two-sample-t-tests. All tests will be performed on a significance level of 0.05 (two-sided). The entire multiple testing procedure keeps the global significance level of 0.05. The primary analysis will be conducted with the intention-to-treat population. Missing values will most likely occur due to patients' death. In this case, no imputation for missing values will be done. If more than 5% of missing values occur due to other reasons multiple imputation for the primary outcome variable will be applied.

We expect that supervised training is most beneficial to the patients. However, we also expect some benefit for patients in the home-based training. If the intervention(s) show efficacy, our study will allow supportive care options for the large group of resected pancreatic cancer patients, thus, indicating a high relevance. As long as supervised training facilities for oncologic patients are not yet widespread throughout Germany the home-based option could be relevant for many patients. Empowered by the expected results of this RCT as well as the increasing understanding that patients from various cancer sites benefit from exercise after diagnosis, it can be expected that training facilities with qualified staff will built up throughout Germany over the next years.

1. Background

1.1 Clinical and epidemiological background on pancreatic cancer

Cancer of the pancreas is the 4th most common cause of cancer death in Germany, both for men and for women. In 2006, 6.729 men and 7,213 women died of pancreatic cancer, and 6,380 men and 6,980 women were newly diagnosed [1]. Pancreatic cancer is generally diagnosed at a late stage. In parallel, pancreatic cancer is characterized by an aggressive tumor growth with early formation of metastases and with limited therapeutical options. Thus, the cancer is almost always fatal, and characterized by dismal 5-year survival rates of 6% for men and 8% for women [1, 2].

However, surgical resection is the basis for potentially curative treatment and can be performed in about 20% of all pancreatic cancer patients today [3, 4]. The 5-year survival in high-volume centers is about 25% [5-7]. Adjuvant chemotherapy is the standard of care and further increases the 5-year-survival and the median survival to up to 30 months [8-10]. Many patients develop a cachectic status during the progression of the disease, and this syndrome accounts for about 80% of deaths in patients with advanced pancreatic cancers [11]. Moreover, the quality of life is dramatically reduced in patients before and shortly after pancreatic resections compared to normal controls, and quality of life only slowly improves after surgery [12].In addition to the psychological distress, predominantly the physical functioning component of quality of life is reduced after a pancreatic resection for pancreatic cancer. Thus, quality of life among pancreatic cancer patients has become a major concern. Exercise and resistance training have been found to effectively improve e.g. quality of life, functional capacity, and muscular strength, and decrease fatigue levels in patients with various cancer types [13-19].

However, there is a dearth of studies for pancreatic cancer, and, to date, no resistance training intervention studies among pancreatic cancer patients have been reported. Only for primary preventive effects on reducing cancer incidence an association of physical activity with pancreatic cancer risk has already been found. A recent meta-analysis on 28 (22 prospective and 6 retrospective) observational studies estimated the relative risk reduction to be about 25-30% when the highest vs. the lowest categories of total or occupational activity were evaluated [20]. For tertiary prevention, there are only case-reports from long-term pancreatic cancer survivors who note vast improvement in overcoming the disease with consistent training (press conference and personal communication, Exercise and Energy Balance Conference (EEBC), 2010, Heidelberg, http://www.eebc2010.com) as well as one recently published study based on a walking intervention (see 4.1.2).

1.2 Randomized controlled trial(s) of exercise among pancreatic cancer patients

Just recently, the only randomized controlled intervention study has been reported, which examined the effects of walking on fatigue levels and quality of life in post-operative pancreatic cancer patients. The authors analyzed data from 110 resected pancreatic cancer patients (n= 102 analyzable) randomized either into a 3-month home-based walking program or a usual care group. Patients were recruited after recovery from pancreatectomy and the exercise group received an instruction-manual based on a graduated walking program including monthly follow-up (reminder) phone calls. Assessments took place at baseline and in a wide range from 3 to 6 month after hospital discharge depending on the end of adjuvant therapy. Regarding fatigue, the authors reported a significant reduction in fatigue scores over time in the walking group (means: 4.8 → 3.5), a non-significant reduction in the usual care group (means: 4.7 → 3.8), and no significant between-group effects. Comparable data were found for quality of life. Standardized assessment methods with regard to physical performance (e.g. cardiopulmonary fitness testing) were lacking. Based on this first

randomized controlled trial it can be assumed that an exercise intervention in pancreatic cancer patients is feasible and suggests benefits. However, it appeared that the applied walking program was not sufficient to induce significant health benefits in the exercise group compared to the usual care group. Based on these first results, randomized controlled and better structured intervention trials with sufficient (supervised) exercise loads are needed to provide strong evidence for beneficial effects of exercise in pancreatic cancer patients.

1.3 Own preliminary work

Expertise and preliminary studies of the investigator team

Heidelberg is uniquely suited for undertaking a randomized controlled trial of exercise among pancreatic cancer patients. The study team consists of experts from the fields of medicine, exercise science, biostatistics, and epidemiology. The Surgical Clinic of the University of Heidelberg is one of the leading institutions for pancreatic surgery in the world. In addition, it runs a major pancreatic cancer research center (basic, translational, and clinical research).The National Center for Tumor Diseases (NCT) is among the German centers with the most experience in undertaking randomized controlled trials as well as epidemiologic studies in the field of exercise and cancer. The NCT team currently conducts exercise trials with breast cancer patients (two RCTs, n=100 patients in NCT01106820, and n=160 inNCT01468766), lung cancer patients (pilot exercise intervention study, n=40) as well as with patients during and up to 1 year after allogeneic stem cell transplantation (n=180, NCT01374399). Our first completed RCT in 105 allogeneic stem-cell transplanted cancer patients has reported substantial improvements in quality of life and physical functioning in this difficult patient group [21]. Furthermore, preliminary analysis in the above mentioned study with advanced lung cancer patients (n= 40) showed that endurance and strength capacity can be enhanced by an individualized exercise program during chemo-/radiotherapy. These findings underline the need for exercise intervention studies in cancer populations who suffered massively from intensive cancer treatment and from the tumor itself.

Preliminary data specific to pancreatic cancer

We have already obtained data and experience regarding the feasibility and safety of this trial in various ways: (1) A survey on 136 patients after pancreatic resections has been performed to evaluate the acceptance of a trial that evaluates the effects of supervised resistance exercise. Overall, 82% (112 patients) of the patients stated that they would be willing to participate in such a trial. (2) There has been previous, preliminary work at the DKFZ and the NCT where pancreatic cancer patients were trained and physiologic (e.g. cachexia) and immunologic parameters were studied [11, 22, 23]. (3) Further pilot data and resistance training experience from 23 pancreatic cancer patients (median age: 60.5 years; 11 female and 12 male) who exercised in a group-based setting at the NCT are available. If possible, cardiorespiratory fitness testing was performed prior to the first training session to detect exercise-relevant cardiac impairments. Tested pancreatic patients reached a mean $VO_{2peak}$of 21.2 ml/kg * min (SD±5.0; Range: 14.3-30.4). Three patients were not able to perform cardiorespiratory fitness testing due to extreme weakness. Six patients living far away only asked for a counseling appointment for developing an individual exercise plan. The goal of the counseling session was to qualify patients for self-administered exercise training near or at patients' home. Fourteen out of 23 patients participated in our supervised resistance training. Mean participation time was 25 weeks (Range: 5-57) with a number of 29 training sessions per patient resulting in a training frequency of 1.3 sessions/week. Eleven out of 14 patients were able to increase training weights within the first 6 training weeks. In 3 cases weights remained stable. No serious adverse events have been reported by any patient, only one patient developed mild to severe pain symptoms during exercise training (but intensity-reduced resistance training was possible). These preliminary training

experiences and data show that resistance training is suitable and feasible in pancreatic cancer patients.

2. Study Design

2.1 Goals of the study / Expected Results

This is a project with high relevance for pancreatic cancer patients, either resectabel or non-resectabel. The major goal of this first RCT is to evaluate the effects of exercise on pancreatic cancer patients on quality of life, fatigue symptoms and physical fitness by comparing

(1) a 6-month supervised moderate-to-high intensity resistance training vs. standard therapy and

(2) a 6-month home-based exercise training vs. standard therapy.

(3) the 6-month supervised progressive resistance training vs. the 6-month home-based exercise training

2.2 Objectives

The primary objective is:

- To investigate the effects of the interventions on the physical functioning subscale of the validated EORTC QLQ-C30 from baseline to the end of the intervention.

Secondary objectives are:

- To investigate the effects of the interventions on overall quality of life from EORTC QLQ-C30 and pancreatic cancer specific module QLQ-PAN26, fatigue, endurance (VO_{2peak}) and strength performance (isometric and isokinetic), adherence to the interventions, discontinuation of adjuvant therapy, body weight and composition, disease progression, overall and progression-free survival.

As global null hypothesis we formulate that the changes in the physical functioning subscale of the quality of life questionnaire EORTC-QLQ-C30 from baseline to 6 months will be the same in the three groups. If global differences are detected, pairwise comparisons between the groups will be made (closed testing procedure as described in 4.4.10). We expect that supervised training is most beneficial to the patients; however, we also expect some benefit for patients in the home-based training.

If the intervention(s) show efficacy, our study will allow supportive care options for pancreatic cancer, which is a major health problem due to its high incidence and mortality. Currently, therapeutical options are very limited, and clinical experience shows that pancreatic patients are among those patients that are, in general, highly motivated to participate in (supportive) therapy options to improve their quality of life. Thus, this study will have realistic practical implications in cancer patients' care. As long as supervised training facilities for oncologic patients are not yet widespread throughout Germany the home-based option could be relevant for many patients. As we increasingly understand that cancer patients from various sites benefit from exercise after diagnosis it can be expected that training facilities with qualified staff will built up over the next years, maybe empowered by the expected results of this RCT.

2.3 Study type / setting

This randomized, controlled three-arm trial investigates the effects of two independent intervention arms: (1) a supervised moderate-to-high resistance training (group EX1) and (2) a home-based exercise program (group EX2) (Figure 1). The two interventions differ primarily by the intensity and mode-of-administration of the intervention (see section 3). It is planned to offer the supervised training program in only one exercise facility, which is located on the Heidelberg University's campus. For feasibility and adherence reasons, we thus plan to recruit only local patients (living within a distance of approx. 20 km) into intervention arm EX1 and only patients who live farther away into intervention arm EX2.
For each patient, the duration of the intervention is 6 months. The minimum follow-up time per patient is 12 months, with further follow-up within the standard patient management of the Surgical Clinic. After the intervention, the participation in one of the two training programs will be offered to all patients irrespective of their group membership for 6 months.

Figure 1: Overview of study design within the first year

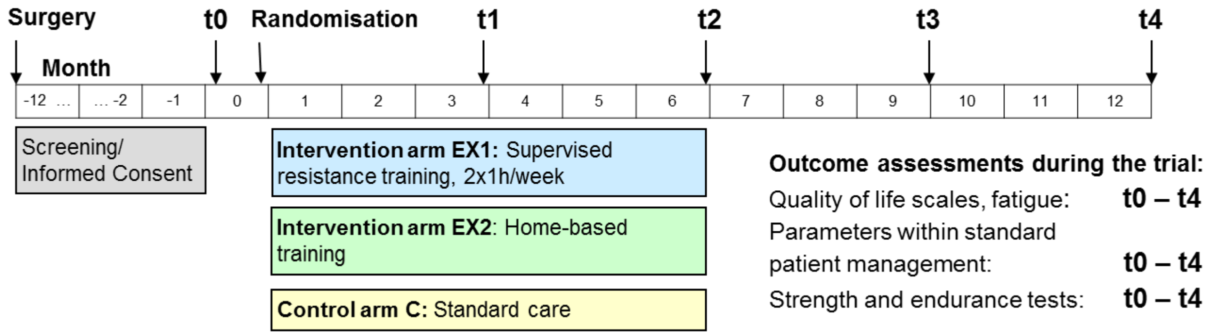

2.4 Outcomes

We aim to develop an exercise intervention program that results in measurable benefits for each pancreatic cancer patient. Thus, we evaluate whether the intervention improves physical functioning, overall quality of life, fatigue, physical performance, and further psychosocial parameters like depression, anxiety and demoralization. Effects will primarily be assessed at the end of the exercise intervention program (timepoint t2, 6 months) and will be adjusted for the baseline values.

Primary efficacy endpoint:

- Physical functioning score, as assessed by EORTC QLQ-C30

Secondary endpoints:

- Overall quality of life from EORTC QLQ-C30 and QLQ-PAN26 (specific module for pancreatic cancer)
- Fatigue (Multidimensional Fatigue Inventory (MFI))
- Endurance (VO_{2peak}), 6 minute walk distance and strength performance (isometric and isokinetic) by ISOMED 2000
- Adherence to the interventions
- Discontinuation and toxicity of adjuvant therapy
- Body weight, height, and body composition
- Disease progression and progression-free survival (time from randomization to either death or disease progression whichever occurs first)
- Overall survival (time from randomization to death)
- Psychosocial parameters (depression, anxiety, demoralization)

- Resting metabolic rate (in a subsample of n= 20)
- Cognition test (Trail Test)
- Biomarkers of cancer risk and progression
- Diet (24h recall)

Furthermore, safety analyses will be based on Serious Adverse Events (SAE´s) and adverse events (AE's) deemed to be causally related to the study interventions.

2.5 Sample size calculation

The primary efficacy analysis compares mean changes between groups in the EORTC QLQ-C30 physical functioning subscale from baseline to the end of the 6-month intervention period based on analysis of covariance. An effect size of 0.6 has recently been recommended for the EORTC-QLQ-C30 physical functioning subscale [24]. Table 1 summarizes different scenarios of sample size calculations, varying by the assumed effect size in intervention group EX2, based on a F-Test comparing normal mean differences of 3 balanced groups with a two-sided significance level of 0.05 (PROC GLMPOWER, SAS version 9.2).

Table 1: Different scenarios for sample size calculation

Assumed effect size in intervention group		Total sample size needed for power=0.8	Power for a total sample size of n=150
EX1	EX2		
0.6	0.4	159	0.78
0.6	0.5	144	0.82
0.6	0.6	126	0.88

Thus, a sample size of 50 evaluable patients per group (n=150 total) in a balanced group design has a power of 82.0% to detect an effect size of 0.6 in one group and of 0.5 in the other. Assuming a maximum drop-out rate of about 25% (primarily due to death, drop-outs due to other reasons have been minimal in our previous exercise trials among cancer patients), 67 patients need to be recruited per group. Thus, the 3-arm trial will allocate a total of 201 patients. Given the number of eligible patients, the total recruitment phase will approximately be 2 years (Figure 2).

2.6 Inclusion and exclusion criteria

We propose to recruit 201 pancreatic cancer patients (stage I-IV). Patients are thus eligible if they meet the following inclusion criteria:

- Pancreatic cancer patients (resectable (stage I-IV) or non-resectable patients)
- Patients ≥ 18 years of age
- Resection performed at the University Clinic of Heidelberg or at a regional cooperation clinic
- Sufficient German language skills
- Signed informed consent

Patients will be excluded from the trial if they meet the following exclusion criteria:

- Heart insufficiency > NYHA III or uncertain arrhythmia
- Uncontrolled hypertension

- Severe renal dysfunction (GFR < 30%, Creatinine > 3mg/dl)
- Bone metastasis in structural relevant areas (e.g. spine)
- Uncompleted wound healing
- Reduced standing or walking ability
- Insufficient hematological capacity (either hemoglobin value below 8 g/dl or thrombocytes below 50.000)
- Any other comorbidities that precludes participation in the exercise

2.7 Randomization procedure

After a baseline visit at the NCT, patients will be randomized to one of the intervention arms, dependent on the living distance from Heidelberg, or the control arm. For both groups, the local patients and the patients who live farther away, a 2:1 randomization scheme will be applied, so that the control group (group C) will consist of an equal number of local patients and patients who live farther away. It is assumed that living distance from Heidelberg is not a predictor for changes in endpoints. Randomization will use block randomization and will be stratified by age, gender and type of treatment (resection and chemotherapy or chemotherapy only). The allocation scheme will be made with a computerized random number generator. Allocation of a patient will be done by an independent biometrician according to the allocation list. Thus, personnel involved in the recruitment and in the baseline assessment will not have any influence on the allocation of the patients, and the biometrician will not be involved in patient recruitment. In this type of study it is not possible to blind participants to the study arm allocation.

Figure 2: Patient flow chart

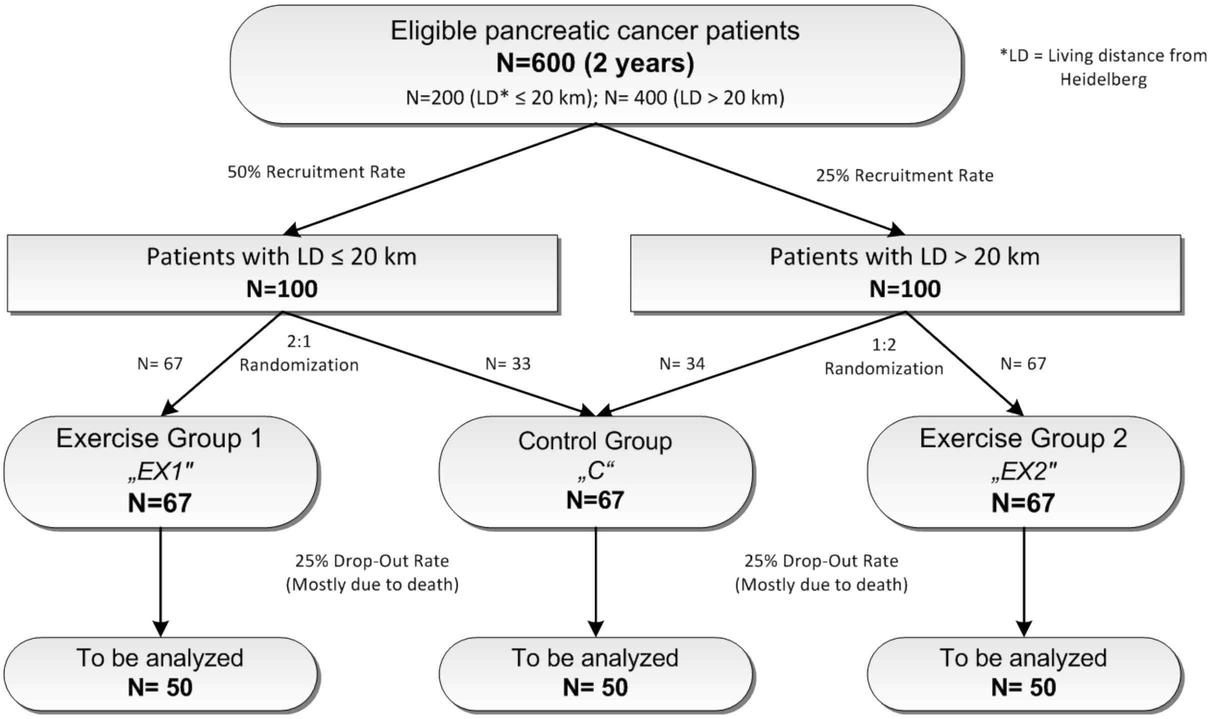

3. Interventions

<u>Intervention group “EX1”</u>: Supervised Resistance Training

The supervised resistance exercise program will take place twice weekly over 6 months in small groups (up to 12 persons per group), guided by the exercise physiotherapist. The resistance training starts with an individual introduction session at the beginning. During the first 4 weeks (month 1 of the intervention) training is planned as a “warm-up” period with exercise intensities ranging from low to moderate. Throughout this warm-up period the patients will perform a one/two-set strength training with a 20-repetition maximum (20 RM, 50-60% of 1-RM) per set. Sessions will comprise resistance exercise machines and free weights. The hypothetic one-repetition maxima according to the Brzycki-Method (1993) will be defined for each machine or exercise task in the second training session [25]. The first session is seen as familiarization work out (familiarization session will be performed to avoid learning effects). The resistance program includes seven exercises for major upper and lower muscle groups: 1) Leg press; 2) Leg extension; 3) Legcurl; 4) Seated row; 5) Shoulder internal rotation; 6) Shoulder external rotation; and 7) Latissimus pull down. A complete session will take approximately 60minutes. Besides setting each weight according to the 1-RM testing all patients will be instructed in using the RPE (rate of perceived exertion) Scale developed by Borg et al [26]. Integrating subjective instruments for tailoring exercise work load in cancer patients is necessary because of variations in patients’ health status, physical fitness and well-being during the treatment period.

The moderate-to-high-intensity progressive resistance training regime starting at month 2 of the intervention period will also be based on the above mentioned 7 exercises. Training will be progressive in that weight will be increased to the next machine weight level (at least by 5%) after successfully completing 3 sets of an exercise with 12 repetitions in three consecutive exercise sessions.

Resistance training set up complies with the American College of Sports Medicine (ACSM) exercise guidelines for cancer survivors [16] and with ACSM recommendations for progressive resistance training for novice weightlifters and older adults for one to three sets at a weight that can be done/lifted for 8 to 12 repetitions (approximately 60–80% of 1-RM) [27, 28]. Resting time between sets will be 1–2 min. The described resistance training is well established at our exercise facility and currently used in our resistance training studies with breast cancer patients.

Intervention group “EX2”: Unsupervised Exercise Training

All patients allocated to the unsupervised intervention group will receive an appointment for an individual face-to-face exercise counseling session at the NCT. During this appointment the patient will receive an exercise manual for individualized home-based exercise and a practical introduction by the physiotherapist. The program combines resistance training (based on own body weight and resistance bands), balance and flexibility exercises. Intervention guidelines will consist of 3 resistance training sessions per week. For non-cachectic patients, 1-2 endurance sessions/week instead resistance training will be allowed. Warm-up and cool-down periods will be 5-10 minutes of light aerobic activity (e.g. walk/rise on tiptoe) and stretching. Strength training will include exercises for the upper and lower extremities with and without resistance (stretch) bands with varying levels of resistance (8-20 repetitions, 2 or 3 sets). Three different strength-training protocols will be used: (1) focused on extremities or (2) the entire body.

Training intensity will be adapted using the RPE scale (Borg) (target scores 14-16 for resistance exercises). Furthermore, patients will be asked to fill in an assessment of pain, fatigue, emotional status, and distress before the exercise session starts. This assessment will be used to self-rate patients’ well-being and group them into 3 different categories (red, yellow, and green) for tailoring the exercise intervention. Green codes for subjective good or normal health status and includes the most challenging exercise recommendations. Yellow and red correspond to medium or bad health status resulting in less challenging recommendations. After each exercise session patients will be asked to document their training in a training log.

If endurance training is clinically allowed, primarily (brisk) walking for 20 to 40 minutes will be recommended (RPE scores between 12 and 14). If patients have experience in Nordic walking or jogging, these techniques will be also recommended. In weekly phone calls, the physiotherapist will review adherence to the intervention and identify problems. This program has been already successfully tested and evaluated in patients undergoing hematopoietic stem cell transplantation [21]. Furthermore, all patients will receive contact information about cancer exercise groups and training facilities near their homes and, in addition, a phone number to contact an exercise therapist in case of questions.

Contraindications for both exercise groups

For both exercise programs, starting a training session will be contraindicated if any of the following symptoms occur: infections, body temperature ≥ 38°C, severe pain, nausea and dizziness, impaired hematopoietic capacity (platelet counts below 20.000/µL or hemoglobin below 8 g/dL). The exercise sessions will be stopped if pain, dizziness, or other contraindications occur. Patients in home-based settings will be instructed correspondingly.

Control Group “C”:

This group will receive regular phone calls (at least once per month) by study personal as well as an individual counseling session (exercise plan, information with regard to implementation intentions and barrier management) after 6 month (t2).

It can be assumed that patients that enroll in this study are interested a priori in physical activity so that there is some chance of drop-ins in the standard care group. However, we do not expect this to be substantial, particularly not with respect to structured resistance training, as the intervention groups will receive. Overall, we do not consider moderate-to-vigorous physical activity such as walking, cycling, or jogging as specifically problematic as long as no systematic intense training is performed.

Furthermore, we will assess physical activity behavior at the beginning (t0),at the end of the intervention period (t2), and 12 months after baseline (t4) via a questionnaire, and, in addition, will objectively assess strength and endurance performance in all three groups. With this procedure we will be able to address the issue of drop-ins later on in the analyses.

4. Plan of investigation

4.1 Screening and recruitment

All patients at the Heidelberg Surgical Clinic or at further cooperation clinics within the region will be screened for eligibility during the visit of the outpatient clinic, as well as prior to and after surgery by the study nurse. Over the last years, the University Clinic Heidelberg has performed about 300 surgeries annually among patients who would have met eligibility criteria for this trial, about 100 local patients and 200 patients who live farther away (Figure 2). Furthermore, non-resectable patients will be screened at the outpatient clinic of the National Center for Tumor Diseases (NCT), whereabouts 70 pancreatic cancer patients per year have received chemotherapeutical treatment. Those patients, who are eligible for the trial will be contacted by study physicians and the study nurse who will explain the details of the study. Eligible patients who agree to participate in the trial and give written informed consent will be scheduled for a baseline assessment (t0) at the NCT prior to the start of the intervention program.

Over the recruitment period of 2 years, the needed number of patients is 100 for each of these two groups as defined by patients’ living distance (Figure 2). This relates to a recruitment rate of 50% for local and 25% for patients who live farther away. As there is a

possibility of over-recruitment of patients who live farther away and as parallel recruitment of both patient groups should be guaranteed, recruitment of patients who live farther away will be closed quarterly after having reached the required group size. Recruitment rates are deemed to be realistic based on the recruitment rates of our ongoing studies (for example: 79.4% in our transplant trial [21], and 64.2% in our lung cancer trial), as well as the clinicians' practical experience that pancreatic cancer patients are, in general, highly motivated to participate in trials. Furthermore, the recently published study of Yeo et al. reported a recruitment rate of 71.5% in pancreatic cancer patients which supports the feasibility of our expected recruitment rate [29].

4.2 Baseline assessment

At the baseline assessment (t0) a full assessment of all outcome measures will be performed. In addition, comprehensive information will be collected on socio-demographics, physical activity history, medical history, concomitant medication, and tumor characteristics. Furthermore, presence of cachexia defined as reduction in self-reported body weight of more than 5% over the last 6 months will be assessed. The baseline assessment of cardiorespiratory fitness with a bicycle ergometer test will provide further information on patients' safety within our trial. This test as well as some of the other assessments will be performed at the NCT being located close to the Surgical Clinic. After the baseline assessment patients will be randomized.

The interventions will commence at the earliest 8 weeks after surgery to allow for appropriate wound healing and regaining of strength and at the latest 12 months after surgery. For the non-resectable patients who will start with the chemotherapy as therapeutical option, the interventions will commence directly after the diagnosis.

4.3 Data collection procedures

All patients will be seen every 3 months as part of the standard patient management in the outpatient clinic at the Surgical Clinic Heidelberg or in the outpatient clinic at the NCT (Figure 1). In addition to the standard workup which includes clinical evaluation, laboratory tests and CT/MRT-investigation, all endpoint assessments of the present trial will be embedded into this scheme. All outcome measures will be assessed at each visit from baseline to month 12 (t0-t4). The collected data of the bicycle ergometer test will be assessed by the sport scientist. If there is an obligation to notify medical findings detected by chance (e.g. abnormalities in the electrocardiogram) the patient will usually be contacted within 10 working days after the study appointment. In addition, information on cancer treatment will be documented in detail over the time course. The study nurse will inform the study coordinator regularly about the dates of the standard workup so that the parallel assessment of information via questionnaires as well as endurance and strength performance can be scheduled.

We intend to follow-up patients and collect patient reported outcomes and data collected within the standard clinical routine an annual basis to investigate maintenance of exercise, longer-term survival and quality of life.

Table 2: Timetable for study-specific outcome assessments

	T0	T1	T2	T3	T4	FU
	Prior to intervention	Mid of intervention (3 months)	End of intervention (6 months)	3 month after intervention (9 months)	6 months after intervention (12 months)	Follow-up every 12 months up to death
Demography	X					
Anamnestic variables, medical history	X					
Health behavior questionnaire	X		X		X	
Cachexia – self-reported	X					X
Concomitant medication (LOG-Form)	X		X			
Standard clinical assessment	X	X	X	X	X	X
Fatigue (MFI questionnaire)	X	X	X	X	X	X
Quality of life (EORTC questionnaires)	X	X	X	X	X	X
Psychosocial status	X	X	X	X	X	X
Cognitive functioning (trail-making test)	X	X	X	X	X	X
Laboratory test	X	X	X	X	X	X
Body composition (BIA)	X	X	X	X	X	
Weight, height	X	X	X	X	X	
Ergometer test	X	X	X		X	
6 minute walk test (6 MWT)	X	X	X	X	X	
Dietary assessment (24h recall)	X	X	X		X	
Muscle capacity (IsoMed)	X	X	X		X	
Accelerometry	X		X			
Training adherence	At each training session					
Safety variables	At each training session					

4.4 Data collection methods

4.4.1 Questionnaires

The following outcome measures will be assessed via questionnaires:

Quality of life based on EORTC QLQ-C30 and EORTC QLQ-PAN26
The physical functioning subscale from the validated EORTC quality-of-life questionnaire (EORTC QLQ-C30, version 3.0) will be assessed as primary endpoint [30, 31]. Further, overall quality of life will be investigated by the EORTC QLQ-C30 and the EORTC QLQ-PAN26 (specific 26-question module for pancreatic cancer, EORTC phase 3 completed) self-administered questionnaires [32]. Both instruments are available in German.

MFI (Multidimensional Fatigue Inventory)
Cancer-related fatigue will be evaluated by the 20-item self-report Multidimensional Fatigue Inventory (MFI), assessing five dimensions: general and physical fatigue, reduced activity, reduced motivation and mental fatigue [33].The MFI has been used to assess fatigue in a variety of cancer patient studies [34, 35], and psychometric properties of this instrument have been classified as satisfying or good [33].

PHQ-4 (Ultra-Brief Patient Health Questionnaire):
The PHQ-4 (Ultra-Brief Patient Health Questionnaire) questionnaire is a short instrument comprising of 4 items to detect the extent of depression and anxiety [36]. It provides sufficient diagnostic accuracy for major depression and can be used as a screening instrument in cancer populations.

DS (Demoralization Scale)
The demoralization scale (DS) is a relatively new developed questionnaire to detect the extent of existential distress in cancer patients [37]. The German version was validated in 2011 and comprises of 24 questions [38]. The results of the validation showed that the concept of demoralization is a useful instrument to describe the different states of existential distress and the individual incapacity to cope effectively with stressful situations.

Locus of control questionnaire
The locus of control questionnaire [39, 40] comprises of 21 items and evaluates the self-efficacy in terms of illness and health. It is widely used in patients with chronic illness and was adapted to cancer patients to measure perceptions of control in cancer patients [39].

Cognition test (trail making test)
To assess cognitive functioning (attention, cognitive flexibility) the trail making test will be applied, a standardized, valid and reliable method from neuropsychological diagnostics [41]. The time needed to combine a logical sequence of number and letters is measured. Norm and reference values are available.

4.4.2 Exercise performance tests and physical function

Cardiorespiratory fitness
Endurance performance (VO_{2peak}) will be measured by performing a bicycle ergometer test with a step protocol (step every 1 minute of 10 watts). The criteria of exhaustion is defined via BORG scale and respiratory ratio (RER)> 1.1. VO_{2peak} will be defined as highest 10-second average during the test. Cardiorespiratory exercise testing is well established in cancer patients and recommendations for testing procedures as well as safety guidelines in clinical trials with cancer populations exist [42]. The procedure will also be used to exclude exercise-contraindicating cardiac impairments. In addition, a 6-minute walk test (6-MWT) will be performed, assessing the number of meters a patient can walk during 6 minutes. This test is an internationally accepted method to assess the endurance performance of patients with chronic diseases.

Strength performance
Isometric (in 4 different joint angle positions) and isokinetic (at 60° angle speed) muscle capacity will be assessed with the Isomed 2000® diagnostic module (isokinetic evaluation and training machine). In addition, handheld dynamometers will used. Reliability and validity of isokinetic dynamometer machines have been reported in several studies [43-45]. The protocol will include testing of representative muscles groups for upper (elbow flexors and extensors) and lower extremity (knee extensors and flexors, hip extensors and flexors) and is currently well established in different ongoing studies in our working group.

Body composition
Body composition of the participants will be estimated with bioelectrical impedance analysis (BIA). This is a quick and non-invasive, scientifically evaluated method, which determines the electrical impedance, or opposition to the flow of an electric current through body tissues to calculate an estimate of total body water, fat-free body mass and body fat [46]. In addition, body weight in light clothing and height will be measured, and the body mass index will be calculated.

Resting metabolic rate
In a subsample of 20 participants (10 of the supervised resistance group and 10 of the control group), resting metabolic rate (RMR) will be measured by means of indirect calorimetry at t0-t2.

Measurements will take place in the morning after restful night's sleep and an overnight (≥12 h) fast. Participants will be instructed to minimize movement after awaking and to refrain from strenuous exercise and alcohol consumption the day before measurements [47]. They will rest in a supine position in a silent, slightly darkened room for 30 min while gas exchange will be assessed using a stationary breath by breath system (Ergostik, Geratherm Respiratory, Bad Kissingen, Germany). Participants will breathe ambient air through a face mask and samples of the expired air will be analyzed.

Oxygen uptake (VO_2), carbon dioxide output (VCO_2) and respiratory exchange ratio (RER) will be averaged over the 10 min period with the lowest values and maximum changes of ±5 % for VO_2 and VCO_2 and ±10 % for RER [48]. RMR will be calculated using the equation by Weir [49]. Percentages of fat and carbohydrate oxidation will be calculated according to Péronnet and Massicotte [50].

RMR will be given in kcal/d, kcal/d/kg body mass and kcal/d/kg fat free mass. Fat and carbohydrate oxidation at rest will be given as percentages of RMR and oxidation rates in g/min.

Accelerometers

To assess the general physical activity behaviour, all patients will wear accelerometers, small devices worn at the hip, for 10 days close to t1 and t2.

4.4.3 Further assessments & documentation

Adherence to interventions / Exercise logs

Adherence to the exercise programs will be monitored continuously. For patients in EX1, training weights and number of repetitions performed will be documented during each session by the patients and checked by the exercise physiotherapist. Patients in EX2 will be asked to fill out a training protocol for each session. In addition, for this group, training adherence will also be reviewed during the weekly telephone calls by the physiotherapist.

All participants in the training groups are asked to report training information according to the FITT criterion (Frequency, Intensity, Time and Type of exercise). Furthermore, patients are asked to document feelings prior and after the training sessions as well as problems/symptoms with regard to the exercise program.

Physical activity in daily living
Physical activity behavior in the domains of commuting activity, leisure time activities such as cycling, walking, and sports, household and occupational activity will be

assessed - intervention program not included- via a standardized and validated questionnaire (SQUASH) in all study groups before (t0, baseline), after 6 months (t2, end of intervention) and at the end of the study after 12 months (t4) [51, 52].

Side effects and clinical documentation
Side effects and adverse events will be systematically documented during the training periods by protocols, in particular those adverse events deemed to be causally related to the study intervention. Furthermore, clinical or therapy relevant changes like e.g. the discontinuation and toxicity of adjuvant or palliative therapy, disease progression (local vs. systemic, as measured during the clinical routine by evaluation of CT/MRT-scans and tumor markers) as well as overall and progression-free survival will be documented and evaluated.

Nutritional status / 24h nutrition recall
Nutritional status will be assessed at t0-t2 and t4 by using the 24h nutrition recall tool "Freiburger Ernährungsprotokoll". Furthermore we will perform the Nutritional Risk Screening (NRS) to identify patients which are on high risk of mal-/undernutrition [53].

4.4.4 Lab methods

The standard clinical patient management at the outpatient clinic at the Surgical Clinic Heidelberg includes several laboratory tests every 3 months. For biomarker analyses, an urine sample and 38ml of blood (2 EDTA à 9ml, 2 serum à 9ml and 2 ml plasma) will be collected by the study nurse, processed, aliquoted, frozen and stored at -80°C by the lab of the Surgical Clinic. Analyses will include tumor markers (e.g. CA 19-9), hemoglobin, glycohemoglobin (HbA1c), nutritional biomarkers (e.g. albumin, cholinesterase, vitamines A, E, and D3, triglycerides, cholesterine, iron, ferritin, magnesium, phosphate) markers of inflammation and oxidative stress (8-oxo-dG).

4.5 Data management and quality control

Data will be collected on case record forms (CRFs) set up with the TELEFORM® system (Cardiff) at the NCT. CRFs will be scanned, verified in the system and written to an Access database. Further plausibility checks will be performed with this database. Data will be stored and analyzed in pseudonymized form. The data manager will be responsible for these tasks.

4.6 Statistical analyses

Besides descriptive methods, inferential statistics will be applied. Missing values will most likely occur due to patients' death. In this case, no imputation for missing values will be done. If more than 5% of the patients drop out due to other reasons replacement of missing values in the primary outcome variable will be based on multiple imputation (Proc MI and Proc MIANALYZE from SAS). If the percentage is less than or equal to 5%, no imputation of missing values will be done.

Each patient's allocation to the different analysis populations (intention-to-treat population, per protocol analysis set) will be defined prior to the analysis. The allocation will be documented in the statistical analysis plan. During the data review, deviations from the protocol will be assessed as "minor" or "major". Major deviations from the protocol will lead to the exclusion of a patient from the per protocol analysis set.

Primary analysis will be based on analysis of covariance (ANCOVA) with intervention group as factor and age and baseline score as continuous covariates. The primary analysis will be

based on the intention-to-treat population. A closed testing procedure will be applied as introduced by Marcus et al. [54]. If the global between-group comparison of all three groups shows significant differences (significance level 0.05), pairwise t-tests (with a significant level of 0.05 each) will be calculated for pairwise comparisons of the groups. The entire multiple testing procedure keeps the global significance level of 0.05. It can be assumed that applying an ANCOVA leads to more power in the analysis of the primary endpoint than the F-test used in the estimation of sample size. Sensitivity analyses of the primary endpoint will include analyses of different populations (per protocol as well as complete case analyses) as well as models with further covariates (e.g. distant to the training facility).

The secondary endpoints will be analyzed descriptively by tabulation of the measures of the empirical distributions. According to the scale level of the variables, means, standard deviations, medians, 1st and 3rd quartiles as well as minimum and maximum or absolute and relative frequencies, respectively, will be reported. Additionally, for the variables with longitudinal measurements (such as quality of life) the time courses of individual patients' data, summarized by group assignment, will be compared. Descriptive p-values of the corresponding statistical tests comparing the intervention groups and associated 95% confidence intervals will be given. If applicable, additional standard time-to-event analyses(Kaplan-Meier-estimates of the survivor curves, log-rank test for differences between the survivor curves, Cox regression models for an exploratory investigation of prognostic factors). will be performed. The homogeneity of the intervention groups will be described by comparison of the demographic data and the baseline values. Further subgroup analyses, for example for cachectic patients, will be performed. Analysis of safety will be based on descriptive analyses of adverse event reporting, in particular of adverse events deemed to be causally related to the study interventions. Adverse events and secondary analyses will be based on the per-protocol population. All analyses will be performed on a significance level of 0.05 (two-sided) and will be implemented in the statistical software package SAS.

5. Potential unexpected side-effects for study participants

5.1 Interventions

Safety and feasibility of parts of the diagnostic and training procedures have been demonstrated in several studies (parts I and II of the POSITIVE study in lung cancer patients) and in our general exercise program for NCT cancer patients with comparably ill patients. However, as this is the first randomized intervention trial on resistance training in pancreatic cancer patients, we will establish a Data Safety Monitoring Committee. Members will represent expertise on pancreatic cancer, internal medicine and statistics.

Resistance training set up complies with the American College of Sports Medicine (ACSM) exercise guidelines for cancer survivors[16]and with ACSM recommendations for progressive resistance training for novice weightlifters and older adults for one to three sets at a weight that can be done/lifted for 8 to 12 repetitions (approximately 60–80% of 1-RM) [27, 28].

5.2 Diagnostic procedures

5.2.1 Questionnaires

No side effects expected.

5.2.2 Exercise performance tests and physical function

Isokinetic muscle testing
Based on the testing procedure patients will be asked to provide maximal voluntary muscle contraction during the assessment on the isokinetic testing machine (Isomed2000). Therefore patients may experience extreme muscle-derived fatigue after the end of testing. Some participants will also report stiffness. Damage to tendons, ligaments, bones and joints is extremely rare and mostly related to unrecognized pre-damage.

Cardiopulmonary Exercise Testing (CPET)
During CPET the patient will be charged to his/her individual maximal aerobic threshold in a bicycle ergometer test. Quite often muscular exhaustion, extreme tachycardia or bradycardia, and sometimes joint pain is reported by tested people. All disturbances are reversible and usually not more available a few minutes after the end of exposure.
Severe dyspnea, cough or exercise-induced asthma can occur but this is rare. Infrequently, strong cyanosis, angina pectoris, headache, dizziness pallor or cold sweats are reported. All mentioned symptoms are classified as stopping criteria for the CPET and usually recover quickly after the abort of the test. To avoid cardiorespiratory complications all participants will be monitored by an electrocardiogram (ECG).

Body composition
No side effects expected as only very low voltage with high frequency (> 10 kHz) is applied.

5.3 Contraindications and abortion criteria

For both exercise programs, starting a training or a testing session will be contraindicated if any of the following symptoms occur:

- infections (body temperature ≥ 38°C)
- severe pain
- nausea and dizziness
- impaired hematopoietic capacity (platelet counts below 20.000/µL or hemoglobin below 8 g/dL)

The <u>exercise sessions</u> will be stopped if pain, dizziness, or other contraindications occur. Patients in home-based settings will be instructed correspondingly.

6. Legal and ethical aspects

6.1 Ethical committee and data privacy

The study plan for SUPPORT has been submitted for review to the ethics committee of the Medical Faculty of Heidelberg prior to patient recruitment. Enrollment into the study will begin upon receiving a positive response by the aforementioned committee. The study will be registered at ClinicalTrials.gov before the first patient will be recruited.
Confidentiality (with regard to the Federal Data Protection Act) of all patient-related data is ensured as all data will be pseudonymously (encrypted) stored and evaluated. A separate log relating original patient data with its respective, encrypted data will be created and appropriately secured by password; only study personal will be granted access to this file.

The procedure is as follows:

The name of the patient, date of birth and address will be recorded in writing and electronically on a patient identification list where each patient will receive an identification number (ID). Information about the health/illness of the study participants collected during the study will be recorded and stored separately from the personal information. Immediately after data collection, the data will be pseudonymously stored via the patient ID. All collected data will remain in secured locations/servers in the National Center for Tumor Diseases (NCT) and the Department of Surgery/University Clinic. The written and documented personal data as well as the illness or health information will be sealed and stored separately from each other. The access to electronically stored data will be password protected.
The handling of patient names and other confidential information follow medical confidentiality laws and the provisions outlined in the Federal Data Protection Act (Bundesdatenschutzgesetz). Data transfer may occur (e.g. in case of a meta-analysis request), but only under encrypted conditions; third parties will *not* be able to access original, unencrypted medical records.

6.2 Informed Consent

Prior to participation in SUPPORT, study staff members will inform each potential participant about all important aspects of the study, including personal rights. If patients agree to the conditions of the study, the study staff will have them sign the informed consent (which will include information regarding the possibility of data transfer under encrypted conditions) before the start of the project. A copy of the patient information and consent declaration form is attached.
The participation within the SUPPORT study is voluntary. Patient approval may be withdrawn at any time, for any reason, and without disadvantages for further medical care. Study data of patient who withdrew will be deleted upon request.

Data will only be processed or analyzed pseudonymously. Patients will be informed orally and in writing as to the nature and scope of the proposed investigation, as well as to the potential forwarding of encrypted study-related medical data. The consent of the patient will be verified by the signature on the consent form.

6.3 Data storage & publication intentions

The responsible investigators commit to archive all documents of SUPPORT study for 15 years.

<u>Use of data and publication rights:</u>
Publications based on data collected in the SUPPORT study must be authorized and carried out in close consultations with the study PIs. This also applies to individual case reports.
After completion of the SUPPORT, a joint publication of the overall results is planned. All PIs and any other persons who provided a significant contribution in the planning, implementation and/or evaluation of the SUPPORT study will be included as authors on that manuscript. It is intended that this manuscript will be submitted to a scientific journal no later than 6 months after the end of the study.

6.4 Insurance

The study is based on the “Eigenversicherungsprinzip”. No specific insurance policy has been issued.

7. References

[1] Robert Koch-Institut. Verbreitung von Krebserkrankungen in Deutschland. Entwicklung der Prävalenzen zwischen 1990 und 2010.Beträge zur Gesundheitsberichterstatung des Bundes. Berlin: RKI; 2010.

[2] Jemal A, Siegel R, Xu J, Ward E. Cancer statistics, 2010. *CA Cancer J Clin* 2010; **60:**277-300.

[3] Adler G, Seufferlein T, Bischoff SC, Brambs HJ, Feuerbach S, Grabenbauer G *et al.* [S3-Guidelines "Exocrine pancreatic cancer" 2007]. *Z Gastroenterol* 2007; **45:**487-523.

[4] Hartwig W, Hackert T, Hinz U, Hassenpflug M, Strobel O, Buchler MW *et al.* Multivisceral resection for pancreatic malignancies: risk-analysis and long-term outcome. *Ann Surg* 2009; **250:**81-87.

[5] Wagner M, Redaelli C, Lietz M, Seiler CA, Friess H, Buchler MW. Curative resection is the single most important factor determining outcome in patients with pancreatic adenocarcinoma. *Br J Surg* 2004; **91:**586-594.

[6] Winter JM, Cameron JL, Campbell KA, Arnold MA, Chang DC, Coleman J *et al.* 1423 pancreaticoduodenectomies for pancreatic cancer: A single-institution experience. *J Gastrointest Surg* 2006; **10:**1199-1210.

[7] Hartwig W, Hackert T, Hinz U, Gluth A, Bergmann F, Strobel O *et al.* Pancreatic cancer surgery in the new millennium: better prediction of outcome. *Ann Surg* 2011; **254:**311-319.

[8] Neoptolemos JP, Stocken DD, Friess H, Bassi C, Dunn JA, Hickey H *et al.* A randomized trial of chemoradiotherapy and chemotherapy after resection of pancreatic cancer. *N Engl J Med* 2004; **350:**1200-1210.

[9] Oettle H, Post S, Neuhaus P, Gellert K, Langrehr J, Ridwelski K *et al.* Adjuvant chemotherapy with gemcitabine vs observation in patients undergoing curative-intent resection of pancreatic cancer: a randomized controlled trial. *JAMA* 2007; **297:**267-277.

[10] Neoptolemos JP, Stocken DD, Bassi C, Ghaneh P, Cunningham D, Goldstein D *et al.* Adjuvant chemotherapy with fluorouracil plus folinic acid vs gemcitabine following pancreatic cancer resection: a randomized controlled trial. *JAMA* 2010; **304:**1073-1081.

[11] Bachmann J, Heiligensetzer M, Krakowski-Roosen H, Buchler MW, Friess H, Martignoni ME. Cachexia worsens prognosis in patients with resectable pancreatic cancer. *J Gastrointest Surg* 2008; **12:**1193-1201.

[12] Pezzilli R, Falconi M, Zerbi A, Casadei R, Valli L, Varale R *et al.* Clinical and patient-reported outcomes after pancreatoduodenectomy for different diseases: a follow-up study. *Pancreas* 2011; **40:**938-945.

[13] Cramp F, Daniel J. Exercise for the management of cancer-related fatigue in adults. *Cochrane Database Syst Rev* 2008;CD006145.

[14] Velthuis MJ, Agasi-Idenburg SC, Aufdemkampe G, Wittink HM. The effect of physical exercise on cancer-related fatigue during cancer treatment: a meta-analysis of randomised controlled trials. *Clin Oncol (R Coll Radiol)* 2010; **22:**208-221.

[15] Cramp F, James A, Lambert J. The effects of resistance training on quality of life in cancer: a systematic literature review and meta-analysis. *Support Care Cancer* 2010; **18:**1367-1376.

[16] Schmitz KH, Courneya KS, Matthews C, Denmark-Wahnefried W, Galvao DA, Pinto BM *et al.* American College of Sports Medicine roundtable on exercise guidelines for cancer survivors. *Med Sci Sports Exerc* 2010; **42:**1409-1426.

[17] Pekmezi DW, Demark-Wahnefried W. Updated evidence in support of diet and exercise interventions in cancer survivors. *Acta Oncol* 2011; **50:**167-178.
[18] Lakoski SG, Eves ND, Douglas PS, Jones LW. Exercise rehabilitation in patients with cancer. *Nat Rev Clin Oncol* 2012.
[19] Brown JC, Huedo-Medina TB, Pescatello LS, Pescatello SM, Ferrer RA, Johnson BT. Efficacy of exercise interventions in modulating cancer-related fatigue among adult cancer survivors: a meta-analysis. *Cancer Epidemiol Biomarkers Prev* 2011; **20:**123-133.
[20] O'Rorke MA, Cantwell MM, Cardwell CR, Mulholland HG, Murray LJ. Can physical activity modulate pancreatic cancer risk? a systematic review and meta-analysis. *Int J Cancer* 2010; **126:**2957-2968.
[21] Wiskemann J, Dreger P, Schwerdtfeger R, Bondong A, Huber G, Kleindienst N *et al.* Effects of a partly self-administered exercise program before, during, and after allogeneic stem cell transplantation. *Blood* 2011; **117:**2604-2613.
[22] Renk, H., Krakowski-Roosen, H., Weber, M. A., Kinscherf, R., Künkele, A., Schmitt, T. L. *et al*. Progressive Resistance Training for resititution of Muscle Mass and Strength in Human Cancer. 11th annual Congress of the European College of Sport Science Lausanne/Switzerland, July 5th-8th, 2006 . 2006.
[23] Künkele, A., Renk, H., Schmitt, T. L., Hildebrandt, W., and Krakowski-Roosen, H. Resistance Training improves Natural Killer Cell Activity in Cachectic Cancer Patients. 11th annual Congress of the European College of Sport Science Lausanne/Switzerland, July 5th-8th . 2006.
[24] Cocks K, King MT, Velikova G, Martyn St-James M, Fayers PM, Brown JM. Evidence-based guidelines for determination of sample size and interpretation of the European Organisation for the Research and Treatment of Cancer Quality of Life Questionnaire Core 30. *J Clin Oncol* 2011; **29:**89-96.
[25] Brzycki M. Strength testing: predicting a one-rep max from reps to fatigue. *Hlth Phys Educ Rec Dance* 1993; **64:**88-90.
[26] Borg G. *Perceived Exertion and Pain Scales*. 1998. Champaign, IL, Human Kinetics.
[27] American College of Sports Medicine position stand. Progression models in resistance training for healthy adults. *Med Sci Sports Exerc* 2009; **41:**687-708.
[28] Chodzko-Zajko WJ, Proctor DN, Fiatarone Singh MA, Minson CT, Nigg CR, Salem GJ *et al.* American College of Sports Medicine position stand. Exercise and physical activity for older adults. *Med Sci Sports Exerc* 2009; **41:**1510-1530.
[29] Yeo TP, Burrell SA, Sauter PK, Kennedy EP, Lavu H, Leiby BE *et al.* A Progressive Postresection Walking Program Significantly Improves Fatigue and Health-Related Quality of Life in Pancreas and Periampullary Cancer Patients. *J Am Coll Surg* 2012.
[30] Aaronson NK, Ahmedzai S, Bergman B, Bullinger M, Cull A, Duez NJ *et al.* The European Organization for Research and Treatment of Cancer QLQ-C30: a quality-of-life instrument for use in international clinical trials in oncology. *J Natl Cancer Inst* 1993; **85:**365-376.
[31] Fayers, PM., Aaronson, NK., Bjordal, K., Curran, D., Groenvold, M, Curran, D. *et al*. EORTC Scoring Manual. 3rd Edition. EORTC Quality of Life Study Group. 2001. Brussels, European Organisation for Research and Treatment of Cancer.
[32] Fitzsimmons D, Johnson CD, George S, Payne S, Sandberg AA, Bassi C *et al.* Development of a disease specific quality of life (QoL) questionnaire module to supplement the EORTC core cancer QoL questionnaire, the QLQ-C30 in patients with pancreatic cancer. EORTC Study Group on Quality of Life. *Eur J Cancer* 1999; **35:**939-941.

[33] Smets EM, Garssen B, Bonke B, de Haes JC. The Multidimensional Fatigue Inventory (MFI) psychometric qualities of an instrument to assess fatigue. *J Psychosom Res* 1995; **39:**315-325.

[34] Schneider RA. Reliability and validity of the Multidimensional Fatigue Inventory (MFI-20) and the Rhoten Fatigue Scale among rural cancer outpatients. *Cancer Nurs* 1998; **21:**370-373.

[35] Jean-Pierre P, Figueroa-Moseley CD, Kohli S, Fiscella K, Palesh OG, Morrow GR. Assessment of cancer-related fatigue: implications for clinical diagnosis and treatment. *Oncologist* 2007; **12 Suppl 1:**11-21.

[36] Lowe B, Wahl I, Rose M, Spitzer C, Glaesmer H, Wingenfeld K *et al.* A 4-item measure of depression and anxiety: validation and standardization of the Patient Health Questionnaire-4 (PHQ-4) in the general population. *J Affect Disord* 2010; **122:**86-95.

[37] Wein S, Sulkes A, Stemmer S. The oncologist's role in managing depression, anxiety, and demoralization with advanced cancer. *Cancer J* 2010; **16:**493-499.

[38] Mehnert A, Scherwath A, Schirmer L, Schleimer B, Petersen C, Schulz-Kindermann F *et al.* The association between neuropsychological impairment, self-perceived cognitive deficits, fatigue and health related quality of life in breast cancer survivors following standard adjuvant versus high-dose chemotherapy. *Patient Educ Couns* 2007; **66:**108-118.

[39] Watson M, Pruyn J, Greer S, van den Borne B. Locus of control and adjustment to cancer. *Psychol Rep* 1990; **66:**39-48.

[40] Schmitt GM, Lohaus A, Salewski C. [Control beliefs and patient compliance: an empirical study exemplified by adolescents with diabetes mellitus, bronchial asthma and alopecia areata]. *Psychother Psychosom Med Psychol* 1989; **39:**33-40.

[41] Reitan RM. The relation of the trail making test to organic brain damage. *J Consult Psychol* 1955; **19:**393-394.

[42] Jones LW, Eves ND, Haykowsky M, Joy AA, Douglas PS. Cardiorespiratory exercise testing in clinical oncology research: systematic review and practice recommendations. *Lancet Oncol* 2008; **9:**757-765.

[43] Drouin JM, Valovich-mcLeod TC, Shultz SJ, Gansneder BM, Perrin DH. Reliability and validity of the Biodex system 3 pro isokinetic dynamometer velocity, torque and position measurements. *Eur J Appl Physiol* 2004; **91:**22-29.

[44] Eitzen I, Hakestad KA, Risberg MA. Inter- and intrarater reliability of isokinetic thigh muscle strength tests in postmenopausal women with osteopenia. *Arch Phys Med Rehabil* 2012; **93:**420-427.

[45] Webber SC, Porter MM. Reliability of ankle isometric, isotonic, and isokinetic strength and power testing in older women. *Phys Ther* 2010; **90:**1165-1175.

[46] Valentinuzzi ME. Bioelectrical impedance techniques in medicine. Part I: Bioimpedance measurement. First section: general concepts. *Crit Rev Biomed Eng* 1996; **24:**223-255.

[47] Speakman JR, Selman C. Physical activity and resting metabolic rate. *Proc Nutr Soc* 2003; **62:**621-634.

[48] Carlsohn A, Scharhag-Rosenberger F, Cassel M, Mayer F. Resting metabolic rate in elite rowers and canoeists: difference between indirect calorimetry and prediction. *Ann Nutr Metab* 2011; **58:**239-244.

[49] Weir JB. New methods for calculating metabolic rate with special reference to protein metabolism. *J Physiol* 1949; **109(1-2):**1-9.

[50] Peronnet F, Massicotte D. Table of nonprotein respiratory quotient: an update. *Can J Sport Sci* 1991; **16:**23-29.

[51] Wendel-Vos GC, Schuit AJ, Saris WH, Kromhout D. Reproducibility and relative validity of the short questionnaire to assess health-enhancing physical activity. *J Clin Epidemiol* 2003; **56:**1163-1169.
[52] Wagenmakers R, van dA-S, I, Groothoff JW, Zijlstra W, Bulstra SK, Kootstra JW *et al.* Reliability and validity of the short questionnaire to assess health-enhancing physical activity (SQUASH) in patients after total hip arthroplasty. *BMC Musculoskelet Disord* 2008; **9:**141.
[53] Kondrup J, Rasmussen HH, Hamberg O, Stanga Z. Nutritional risk screening (NRS 2002): a new method based on an analysis of controlled clinical trials. *Clin Nutr* 2003; **22:**321-336.
[54] Marcus R, Peritz E, Gabriel KR. On closed testing procedures with special reference to ordered analysis of variance. *Biometrika* 1976; **63:**655-660.

8. Attachments

Patient's information for patients with a living distance of not more than 20 km from the study center (in German) page 27

Patient's information for patients with a living distance of less than 20 km from the study (in German) page 32

Informed consent (in German) page 37

Page of signatures for approval of the study protocol page 38

Version Fernpatient

Patienteninformation

zur

SUPPORT - Studie

(**Sup**ervidiertes, **pro**gressives K**r**aft**t**raining für Pankreaskarzinompatienten und -innen: eine randomisierte, kontrollierte Interventionsstudie)

Patient: ______________________, ______________________
(Name) (Vorname)

Sehr geehrte Patientin, sehr geehrter Patient,

wir laden Sie ein, an der nachfolgend beschriebenen Studie teilzunehmen.

Zweck der Studie

Bei Ihnen wurde eine Krebserkrankung der Bauchspeicheldrüse diagnostiziert. Nicht selten treten im Verlauf der Therapie körperliche Einschränkungen und damit einhergehend starke Erschöpfungssymptomatiken auf.

Wissenschaftliche Untersuchungen bei anderen Tumorarten deuten darauf hin, dass körperliches Training genau wie bei gesunden Personen zu einer Steigerung der körperlichen Fitness und damit zur Verbesserung des Wohlbefindens beitragen kann. Zudem werden möglicherweise krankheits- und therapiebedingte Nebenwirkungen wie die angesprochene Erschöpfungssymptomatik gelindert und damit die Lebensqualität positiv beeinflusst. Im Rahmen der SUPPORT-Studie wollen wir daher herausfinden, welche Effekte durch körperliches Training bei Patienten/-innen mit Bauchspeicheldrüsenkrebs erzielt werden können. Um dies untersuchen zu können, werden einige Teilnehmer, zufällig durch Losverfahren bestimmt, eine bewegungstherapeutische Beratung bzw. Betreuung mit individuell angepassten Übungen zu erhalten.

An dieser Studie werden ungefähr 200 Patienten mit Bauchspeicheldrüsenkrebs hier in Heidelberg teilnehmen. Die finanzielle Förderung hat die Deutsche Krebshilfe e.V. übernommen.

Freiwilligkeit

Ihre Teilnahme an diesem Projekt ist freiwillig. Sie können selbst entscheiden, ob Sie daran teilnehmen wollen.

Wenn Sie sich für die Teilnahme entscheiden, unterschreiben Sie bitte die beiden Seiten am Ende dieses Formblatts. Damit zeigen Sie, dass Sie einverstanden sind, an dieser Studie teilzunehmen.

Sie können während der Studie jederzeit Ihre Meinung ändern und aus der Studie ausscheiden, auch wenn Sie dieses Formblatt unterschrieben haben. Ihnen entstehen dadurch keine Nachteile in der weiteren ärztlichen Versorgung und Sie müssen dafür keine Gründe angeben. Ihre im Zusammenhang mit der Studie erhobenen Daten und Proben können auf Ihren Wunsch in diesem Fall vernichtet werden.

Bitte beachten Sie:

Um die Auswirkungen von körperlichem Training untersuchen zu können, umfasst die Studie drei Vergleichsgruppen, die erste Gruppe erhält keine zusätzliche Behandlung, die zweite Gruppe ein therapeutenangeleitetes Krafttraining an unserem Studienstandort Heidelberg, die dritte Gruppe ein zu Hause durchzuführendes Krafttraining gemäß einem individuell für Sie erarbeiteten Trainingsplan. Die Zuordnung in die Gruppen 2 oder 3 richtet sich nach Ihrem Wohnort. Nur Personen, die höchstens 20 km vom Studienstandort Heidelberg entfernt wohnen, können in die Gruppe 2 gelost werden. Ebenso können nur Personen, die mehr als 20 km entfernt wohnen, in die Gruppe 3 aufgenommen werden. Die Zuweisung zur Kontrollgruppe erfolgt unabhängig vom Wohnort.

Da Sie mehr als ca. 20 km vom Studienzentrum entfernt wohnen, würden Sie bei Studienteilnahme zufällig der zu Hause trainierenden Gruppe oder der Kontrollgruppe zugeordnet werden, mit einer Zuteilungsquote von 2:1.

Wie lange dauert die Studienteilnahme?

Ihre aktive Teilnahme an der Studie wird 6 Monate betragen, gefolgt von einer 6-monatigen Nachbeobachtungszeit.

Weiterhin sind wir an Ihrem langfristigen Wohlbefinden interessiert und würden Sie im jährlichen Abstand bitten (bis zu max. 6 Jahre), Fragebögen zu Ihrem Befinden auszufüllen.

Ablauf der Studie

Sie werden von Ihrem behandelnden Arzt angesprochen, ob Sie an dem Projekt teilnehmen möchten. Nachdem Sie Ihre Zustimmung erteilt haben, vereinbart unser Studienpersonal mit Ihnen einen für Sie passenden Termin. An diesem werden erstmals die verschiedenen Faktoren erfasst, die zur Beantwortung der wissenschaftlichen Fragestellungen nötig sind. Eine Untersuchung dauert maximal 3 Stunden.

Anschließend werden Sie einer der beiden für Sie in Frage kommenden Gruppen zufällig zugeordnet. Wurden Sie der Trainingsgruppe zugewiesen, beginnen Sie ca. 10 Wochen bis maximal 12 Monate nach Ihrer Operation mit dem 6-monatigenTraining. Vor Ihrem Trainingsbeginn findet zeitnah die Trainingseinführung am Studienzentrum statt, für die wir 1 ½ Stunden benötigen.

Um die Wirksamkeit des körperlichen Trainings wissenschaftlich evaluieren zu können, werden alle 3 Monate diagnostische Untersuchungen über einen Gesamtzeitraum von einem Jahr wiederholt. Diese werden zeitlich in den Rhythmus Ihrer üblichen klinischen Untersuchungsroutinen eingebettet, so dass in der Regel keine zusätzlichen Anreisen Ihrerseits nötig sein sollten.

Die Studienuntersuchungen umfassen eine schriftliche Befragung (ca. 20 Minuten), einen Test der Ausdauerleistungsfähigkeit auf einem Fahrradergometer (ca. 30 Minuten) und einen Test der Muskelfunktion mittels Krafttestmaschine (ca. 50 Minuten). Alle Tests finden in der Chirurgischen Klinik oder im Nationalen Centrum für Tumorerkrankungen (Im Neuenheimer

Feld 460, Heidelberg). Für beide Standorte sind Bushaltestellen sowie Parkplätze vorhanden. Zudem werden eine Urin- und eine Blutprobe (max. 38 ml) genommen. Ihre Proben werden eingefroren und für die Analysen nach Studienabschluss des/r letzten Teilnehmers/-in aufbewahrt. Die Proben werden nur im Rahmen studienbezogener Fragestellungen verwendet und nach spätestens 20 Jahren vernichtet. Bei dem Ausdauerleistungstest wird keine sportmedizinische Befundung durchgeführt. Zufallsbefunde im Rahmen des Ausdauerleistungstests werden Ihnen mitgeteilt, sofern sie klinisch relevant sind (z.B. Auffälligkeiten im Belastungs-EKG wie höhergradige Herzrhythmusstörungen). Zusätzlich werden Sie an 2 Zeitpunkten gebeten, für 10 Tage ein kleines Gerät zu tragen, das Daten zu Ihrem Aktivitätsverhalten erfasst.

Während der ersten 6 Monate werden wir Sie ca. 1-2 Mal pro Monat telefonisch zu Ihrer aktuellen Gesundheitssituation befragen. Unabhängig von Ihrer Gruppenzugehörigkeit bieten wir Ihnen im Anschluss an die 6-monatige Trainingsphase die Möglichkeit der kostenfreien Teilnahme am Trainingsprogramm für 6 Monate an.

Welcher Gruppe werden Sie zugeordnet?

Welches Programm Sie in den ersten 6 Monaten erhalten wird ausgelost. Die Studienleiter bzw. -ärzte haben keinen Einfluss darauf, welcher Gruppe Sie zugelost werden.

Mit Ihrer Teilnahme verpflichten Sie sich, während der Studienphase kein zusätzliches systematisches Krafttraining zu absolvieren.

Informationen zum Krafttraining

Sie werden das **Krafttraining** zu Hause oder in Nähe Ihres Wohnorts durchführen. Zu Beginn des Trainings erhalten Sie eine individuelle Beratung und eine praktische Einweisung in die vorgesehenen Übungen durch einen erfahrenen Sport-/Physiotherapeuten. Außerdem erhalten Sie ein schriftliches Trainings-Manual. Die Kraftbelastung Ihres Trainings wird dabei individuell auf Sie eingestellt. Das Studienpersonal steht Ihnen bei Fragen und Problemen telefonisch gerne zur Verfügung. Durch das Training soll Ihre Muskulatur gestärkt werden bzw. neue Muskulatur aufgebaut werden. Dies verspricht für Ihren Alltag eine bessere Beweglichkeit und körperliche Leistungsfähigkeit. Zudem lassen aktuelle Studien zu Krafttraining bei Krebspatienten, eine Verringerung von Fatigue und eine gesteigerte Lebensqualität vermuten.

Wie oft, wie lange und wo wird trainiert?

Das Trainingsprogram erfolgt über einen Zeitraum von 6 Monaten dreimal pro Woche und dauert jeweils 60 Minuten.

Mögliche Risiken, Beschwerden und Begleiterscheinungen

Körperliches Ausdauer- und Krafttraining wurde bereits mit Krebspatienten durchgeführt und es sind bisher keine negativen Auswirkungen bekannt.

Durch das körperliche Training können Anpassungsreaktionen des Körpers auftreten, die ggf. zu leichten Schmerzen (bspw. durch Muskelkater) führen können. Dies gilt auch für die Testungen im Rahmen der körperlichen Untersuchung. Ein Muskelkater beeinträchtigt Sie vorübergehend in Ihrer normalen Beweglichkeit, ist aber nach wenigen Tagen wieder verschwunden. Die diagnostischen Untersuchungen bergen ein extrem geringes Gesundheitsrisiko, das durch medizinische Überwachung weiter minimiert wird.

Die Blutabnahme erfolgt im ärztlichen Standardverfahren aus einer Cubitalvene. Dabei kann es extrem selten zu einem Spritzenabszess, Absterben von Gewebe, oder einer Venenreizung kommen. Selten treten Hämatome auf, die sich regional auf die Einstichstelle beschränken und innerhalb weniger Tage verschwinden. Die entnommene Blutmenge ist so gering, dass nicht mit Kreislaufbeeinträchtigungen zu rechnen ist. Trotzdem kann es in Einzelfällen zu Unwohlsein kommen.

Möglicher Nutzen aus Ihrer Teilnahme an der Studie

Mit der Teilnahme an der Studie können möglicherweise Ihre Beschwerden und Komplikationen während ihrer Behandlung positiv beeinflusst werden. Es ist jedoch auch möglich, dass Sie durch Ihre Teilnahme an dieser Studie keinen direkten Nutzen für Ihre Gesundheit und Ihr Wohlbefinden haben. Die Ergebnisse dieser Studie können jedoch dazu beitragen, dass für andere Patienten eine wirksame Behandlung gefunden wird.

Zusammenfassend: Vor dem Hintergrund möglicher geringer körperlicher Beeinträchtigungen durch die Intervention sowie den geringen Risiken, die durch die Vor- und Nachuntersuchungen entstehen, ist der Nutzen höher als die Risiken einzuschätzen.

Bekomme ich eine Aufwandsentschädigung?

Sie bekommen kein Honorar für die Teilnahme an der Studie. Fahrtkosten zu den diagnostischen Studienuntersuchungen zu den jeweiligen Messzeitpunkten können Ihnen mit 0.30 €/km bzw. bis maximal 10 € pro Messzeitpunkt erstattet werden, sofern diese nicht an Ihren normalen Klinikterminen stattfinden.

Datenschutz

Die im Rahmen der Studie erhobenen Angaben über Ihre Person, sowie Ihre Gesundheit bzw. Krankheit werden von den im Projekt tätigen Mitarbeitern getrennt von Ihren persönlichen Angaben handschriftlich und elektronisch aufgezeichnet und sofort nach Erhebung mit einer Kennziffer versehen, die nur den betreffenden Mitarbeitern eine Zuordnung der Krankheits- bzw. Gesundheitsdaten zu Ihrer Person ermöglicht. Dieses Verfahren verläuft wie folgt: Ihr Name wird auf dieser Patienten-Information und Ihrer Patienten-Einwilligung notiert. Zusätzlich werden Geburtsdatum, Ihre Anschrift und Telefonnummern auf einer Patienten-Identifikationsliste schriftlich sowie auch elektronisch dokumentiert. Getrennt davon werden Ihre mit einer Kennziffer versehenen Krankheits- bzw. Gesundheitsdaten erfasst und verarbeitet. All diese Angaben verbleiben getrennt voneinander und separat verschlossen beim Nationalen Centrum für Tumorerkrankungen (NCT). Durch diese Pseudonymisierung können Ihre Daten verarbeitet werden, ohne dass Sie persönlich dabei mit Ihrem Namen erkennbar würden.

Die Ergebnisse der Studie werden daher ohne jede Bezugsmöglichkeit auf Ihre Person voraussichtlich in medizinischen Fachzeitschriften veröffentlicht. Die Beachtung des Bundesdatenschutzgesetzes ist in vollem Umfange sichergestellt.

Pseudonymisierte Proben und Daten können vom Nationalen Centrum für Tumorerkrankungen an nationale oder internationale Kooperationspartner weitergegeben werden, dort für wissenschaftliche Projekte ausgewertet und für zukünftige wissenschaftliche Fragestellungen gelagert werden. Die Proben und Daten werden nur im Rahmen wissenschaftlicher Projekte verwendet und nach spätestens 20 Jahren vernichtet. Durch die

Weitergabe der Daten und Proben an wissenschaftliche Kooperationspartner und Forschungsverbünde haben Sie weder eine Belastung noch persönliche Vorteile. Aber durch die Bündelung von Informationen werden sich weitere Themen erforschen lassen, die wir alleine mit der SUPPORT-Studie nicht beantworten können.

Sie haben das Recht, Auskunft über die Sie betreffenden aufgezeichneten Angaben und die Ergebnisse Ihrer Untersuchung bzw. Behandlung zu verlangen, soweit dies nicht aus technischen Gründen niemandem mehr möglich ist. Sollten Sie einer Weiterverarbeitung Ihrer Daten widersprechen, werden keine weiteren Daten über Ihre Person zum Zweck der o. g. Studie erhoben und aufgezeichnet. Sie können bei unrichtiger Aufzeichnung von Angaben, die Ihre Person betreffen, auch eine Berichtigung dieser Angaben verlangen.

Ihre **Ansprechpartner** für Fragen und Informationen im Rahmen der Studie sind:

Dorothea Frevel
Nationales Centrum für Tumorerkrankungen
Tel.: 06221 – 56 35730
E-mail: Dorothea.Frevel@nct-heidelberg.de

Dr. Christine Tjaden
Chirurgische Klinik, Heidelberg
Tel.: 06221 – 56 39694
E-mail: Christine.Tjaden@med.uni-heidelberg.de

Studienleiter:

Prof Dr. Karen Steindorf
Tel.: 06221-42-2351 und -56-5838
Email: karen.steindorf@nct-heidelberg.de

Prof. Dr. Thilo Hackert
Tel.: 06221-56-5950
Email: thilo.hackert@med.uni-heidelberg.de

Dr. Joachim Wiskemann
Tel.: 06221-56-5904
Email: joachim.wiskemann@nct-heidelberg.de

Prof. Dr. Cornelia Ulrich
Tel: 06221-56 5528
Email: neli.ulrich@nct-heidelberg.de

Prof. Dr. Karen Steindorf

Prof. Dr. Thilo Hackert

Dr. Joachim Wiskemann

Prof. Dr. Cornelia Ulrich

Version Nahpatient

Patienteninformation

zur

SUPPORT - Studie

(**Sup**ervidiertes, **pro**gressives K**r**af**t**training für Pankreaskarzinompatienten und -innen: eine randomisierte, kontrollierte Interventionsstudie)

Patient: ______________________, ______________________
(Name) (Vorname)

Sehr geehrte Patientin, sehr geehrter Patient,

wir laden Sie ein, an der nachfolgend beschriebenen Studie teilzunehmen.

Zweck der Studie

Bei Ihnen wurde eine Krebserkrankung der Bauchspeicheldrüse diagnostiziert. Nicht selten treten im Verlauf der Therapie körperliche Einschränkungen und damit einhergehend starke Erschöpfungssymptomatiken auf.

Wissenschaftliche Untersuchungen bei anderen Tumorarten deuten darauf hin, dass körperliches Training genau wie bei gesunden Personen zu einer Steigerung der körperlichen Fitness und damit zur Verbesserung des Wohlbefindens beitragen kann. Zudem werden möglicherweise krankheits- und therapiebedingte Nebenwirkungen wie die angesprochene Erschöpfungssymptomatik gelindert und damit die Lebensqualität positiv beeinflusst. Im Rahmen der SUPPORT-Studie wollen wir daher herausfinden, welche Effekte durch körperliches Training bei Patienten/-innen mit Bauchspeicheldrüsenkrebs erzielt werden können. Um dies untersuchen zu können, werden einige Teilnehmer, zufällig durch Losverfahren bestimmt, eine bewegungstherapeutische Beratung bzw. Betreuung mit individuell angepassten Übungen zu erhalten.

An dieser Studie werden ungefähr 200 Patienten mit Bauchspeicheldrüsenkrebs hier in Heidelberg teilnehmen. Die finanzielle Förderung hat die Deutsche Krebshilfe e.V. übernommen.

Freiwilligkeit

Ihre Teilnahme an diesem Projekt ist freiwillig. Sie können selbst entscheiden, ob Sie daran teilnehmen wollen.

Wenn Sie sich für die Teilnahme entscheiden, unterschreiben Sie bitte die beiden Seiten am Ende dieses Formblatts. Damit zeigen Sie, dass Sie einverstanden sind, an dieser Studie teilzunehmen.

Sie können während der Studie jederzeit Ihre Meinung ändern und aus der Studie ausscheiden, auch wenn Sie dieses Formblatt unterschrieben haben. Ihnen entstehen dadurch keine Nachteile in der weiteren ärztlichen Versorgung und Sie müssen dafür keine Gründe angeben. Ihre im Zusammenhang mit der Studie erhobenen Daten und Proben können auf Ihren Wunsch in diesem Fall vernichtet werden.

Bitte beachten Sie:

Um die Auswirkungen von körperlichem Training untersuchen zu können, umfasst die Studie drei Vergleichsgruppen, die erste Gruppe erhält keine zusätzliche Behandlung, die zweite Gruppe ein therapeutenangeleitetes Krafttraining an unserem Studienstandort Heidelberg, die dritte Gruppe ein zu Hause durchzuführendes Krafttraining gemäß einem individuell für Sie erarbeiteten Trainingsplan. Die Zuordnung in die Gruppen 2 oder 3 richtet sich nach Ihrem Wohnort. Nur Personen, die höchstens 20 km vom Studienstandort Heidelberg entfernt wohnen, können in die Gruppe 2 gelost werden. Ebenso können nur Personen, die mehr als 20 km entfernt wohnen, in die Gruppe 3 aufgenommen werden. Die Zuweisung zur Kontrollgruppe erfolgt unabhängig vom Wohnort.

Da Sie in etwa 20 km vom Studienzentrum entfernt wohnen, würden Sie bei Studienteilnahme zufällig der therapeutenangeleiteten Krafttrainings- oder der Kontrollgruppe zugeordnet werden mit einer Zuteilungsquote von 2:1.

Wie lange dauert die Studienteilnahme?

Ihre aktive Teilnahme an der Studie wird 6 Monate betragen, gefolgt von einer 6-monatigen Nachbeobachtungszeit.

Weiterhin sind wir an Ihrem langfristigen Wohlbefinden interessiert und würden Sie im jährlichen Abstand bitten (bis zu max. 6 Jahre), Fragebögen zu Ihrem Befinden auszufüllen.

Ablauf der Studie

Sie werden von Ihrem behandelnden Arzt angesprochen, ob Sie an dem Projekt teilnehmen möchten. Nachdem Sie Ihre Zustimmung erteilt haben, vereinbart unser Studienpersonal mit Ihnen einen für Sie passenden Termin. An diesem werden erstmals die verschiedenen Faktoren erfasst, die zur Beantwortung der wissenschaftlichen Fragestellungen nötig sind. Eine Untersuchung dauert maximal 3 Stunden.

Anschließend werden Sie einer der beiden für Sie in Frage kommenden Gruppen zufällig zugeordnet. Wurden Sie der Trainingsgruppe zugewiesen, beginnen Sie ca. 10 Wochen bis maximal 12 Monate nach Ihrer Operation mit dem 6-monatigenTraining. Vor Ihrem Trainingsbeginn findet zeitnah die Trainingseinführung am Studienzentrum statt, für die wir 1 ½ Stunden benötigen.

Um die Wirksamkeit des körperlichen Trainings wissenschaftlich evaluieren zu können, werden alle 3 Monate diagnostische Untersuchungen über einen Gesamtzeitraum von einem Jahr wiederholt. Diese werden zeitlich in den Rhythmus Ihrer üblichen klinischen Untersuchungsroutinen eingebettet, so dass in der Regel keine zusätzlichen Anreisen Ihrerseits nötig sein sollten.

Die Studienuntersuchungen umfassen eine schriftliche Befragung (ca. 20 Minuten), einen Test der Ausdauerleistungsfähigkeit auf einem Fahrradergometer (ca. 30 Minuten) und einen Test der Muskelfunktion mittels Krafttestmaschine (ca. 50 Minuten). Alle Tests finden in der

Chirurgischen Klinik oder im Nationalen Centrum für Tumorerkrankungen (Im Neuenheimer Feld 460, Heidelberg). Für beide Standorte sind Bushaltestellen sowie Parkplätze vorhanden. Zudem werden eine Urin- und eine Blutprobe (max. 38 ml) genommen. Ihre Proben werden eingefroren und für die Analysen nach Studienabschluss des/r letzten Teilnehmers/-in aufbewahrt. Die Proben werden nur im Rahmen studienbezogener Fragestellungen verwendet und nach spätestens 20 Jahren vernichtet. Bei dem Ausdauerleistungstest wird keine sportmedizinische Befundung durchgeführt. Zufallsbefunde im Rahmen des Ausdauerleistungstests werden Ihnen mitgeteilt, sofern sie klinisch relevant sind (z.B. Auffälligkeiten im Belastungs-EKG wie höhergradige Herzrhythmusstörungen). Zusätzlich werden Sie an 2 Zeitpunkten gebeten, für 10 Tage ein kleines Gerät zu tragen, das Daten zu Ihrem Aktivitätsverhalten erfasst.

Während der ersten 6 Monate werden wir Sie ca. 1-2 Mal pro Monat telefonisch zu Ihrer aktuellen Gesundheitssituation befragen. Unabhängig von Ihrer Gruppenzugehörigkeit bieten wir Ihnen im Anschluss an die 6-monatige Trainingsphase die Möglichkeit der kostenfreien Teilnahme am Trainingsprogramm für 6 Monate an.

Welcher Gruppe werden Sie zugeordnet?

Welches Programm Sie in den ersten 6 Monaten erhalten wird ausgelost. Die Studienleiter bzw. -ärzte haben keinen Einfluss darauf, welcher Gruppe Sie zugelost werden.

Mit Ihrer Teilnahme verpflichten Sie sich, während der Studienphase kein zusätzliches systematisches Krafttraining zu absolvieren.

Informationen zum Krafttraining

Das **Krafttraining** findet mit Hilfe verschiedener Trainingsgeräte statt. Die Kraftbelastung ist dabei individuell auf Sie eingestellt. Dabei stellen die ersten 4 Wochen eine Eingewöhnungszeit dar, in der Sie mit leichter bis moderater Intensität trainieren. Erfahrene Sport-/Physiotherapeuten stehen zur Anleitung und Betreuung während des Trainings immer zur Verfügung. Durch das spezielle Krafttraining soll Ihre Muskulatur gestärkt werden bzw. neue Muskulatur aufgebaut werden. Dies verspricht für Ihren Alltag eine bessere Beweglichkeit und körperliche Leistungsfähigkeit. Zudem lassen aktuelle Studien zu Krafttraining bei Krebspatienten, eine Verringerung von Fatigue und eine gesteigerte Lebensqualität vermuten.

Wie oft, wie lange und wo wird trainiert?

Das Trainingsprogram erfolgt über einen Zeitraum von 6 Monaten zweimal pro Woche und dauert jeweils 45-60 Minuten. Sie werden dabei gemeinsam mit anderen Krebspatienten trainieren. Trainingszeiten können individuell gewählt werden. Montag-, Dienstag-, Donnerstag-, Freitag- oder Samstagvormittag stehen zur Auswahl.

Das Training findet am Institut für Sport und Sportwissenschaft der Universität Heidelberg, Im Neuenheimer Feld 720 (Nähe Tiergartenschwimmbad) statt, welches in unmittelbarer Nähe des Nationalen Centrums für Tumorerkrankungen liegt. Bushaltestelle sowie Parkplätze sind vorhanden.

Mögliche Risiken, Beschwerden und Begleiterscheinungen

Körperliches Ausdauer- und Krafttraining wurde bereits mit Krebspatienten durchgeführt und es sind bisher keine negativen Auswirkungen bekannt.

Durch das körperliche Training können Anpassungsreaktionen des Körpers auftreten, die ggf. zu leichten Schmerzen (bspw. durch Muskelkater) führen können. Dies gilt auch für die Testungen im Rahmen der körperlichen Untersuchung. Ein Muskelkater beeinträchtigt Sie vorübergehend in Ihrer normalen Beweglichkeit, ist aber nach wenigen Tagen wieder verschwunden. Die diagnostischen Untersuchungen bergen ein extrem geringes Gesundheitsrisiko, das durch medizinische Überwachung weiter minimiert wird.

Die Blutabnahme erfolgt im ärztlichen Standardverfahren aus einer Cubitalvene. Dabei kann es extrem selten zu einem Spritzenabszess, Absterben von Gewebe, oder einer Venenreizung kommen. Selten treten Hämatome auf, die sich regional auf die Einstichstelle beschränken und innerhalb weniger Tage verschwinden. Die entnommene Blutmenge ist so gering, dass nicht mit Kreislaufbeeinträchtigungen zu rechnen ist. Trotzdem kann es in Einzelfällen zu Unwohlsein kommen.

Möglicher Nutzen aus Ihrer Teilnahme an der Studie

Mit der Teilnahme an der Studie können möglicherweise Ihre Beschwerden und Komplikationen während ihrer Behandlung positiv beeinflusst werden. Es ist jedoch auch möglich, dass Sie durch Ihre Teilnahme an dieser Studie keinen direkten Nutzen für Ihre Gesundheit und Ihr Wohlbefinden haben. Die Ergebnisse dieser Studie können jedoch dazu beitragen, dass für andere Patienten eine wirksame Behandlung gefunden wird.

Zusammenfassend: Vor dem Hintergrund möglicher geringer körperlicher Beeinträchtigungen durch die Intervention sowie den geringen Risiken, die durch die Vor- und Nachuntersuchungen entstehen, ist der Nutzen höher als die Risiken einzuschätzen.

Bekomme ich eine Aufwandsentschädigung?

Sie bekommen kein Honorar für die Teilnahme an der Studie. Fahrtkosten zu den diagnostischen Studienuntersuchungen, sofern diese nicht an Ihrem normalen Klinikterminen stattfinden und zum Training am Institut für Sport und Sportwissenschaften können Ihnen mit 0.30 €/km bzw. maximal 10 € pro Training/Termin erstattet werden.

Datenschutz

Die im Rahmen der Studie erhobenen Angaben über Ihre Person, sowie Ihre Gesundheit bzw. Krankheit werden von den im Projekt tätigen Mitarbeitern getrennt von Ihren persönlichen Angaben handschriftlich und elektronisch aufgezeichnet und sofort nach Erhebung mit einer Kennziffer versehen, die nur den betreffenden Mitarbeitern eine Zuordnung der Krankheits- bzw. Gesundheitsdaten zu Ihrer Person ermöglicht. Dieses Verfahren verläuft wie folgt: Ihr Name wird auf dieser Patienten-Information und Ihrer Patienten-Einwilligung notiert. Zusätzlich werden Geburtsdatum, Ihre Anschrift und Telefonnummern auf einer Patienten-Identifikationsliste schriftlich sowie auch elektronisch dokumentiert. Getrennt davon werden Ihre mit einer Kennziffer versehenen Krankheits- bzw. Gesundheitsdaten erfasst und verarbeitet. All diese Angaben verbleiben getrennt voneinander und separat verschlossen beim Nationalen Centrum für Tumorerkrankungen (NCT). Durch diese Pseudonymisierung können Ihre Daten verarbeitet werden, ohne dass Sie persönlich dabei mit Ihrem Namen erkennbar würden.

Die Ergebnisse der Studie werden daher ohne jede Bezugsmöglichkeit auf Ihre Person voraussichtlich in medizinischen Fachzeitschriften veröffentlicht. Die Beachtung des Bundesdatenschutzgesetzes ist in vollem Umfange sichergestellt.

Pseudonymisierte Proben und Daten können vom Nationalen Centrum für Tumorerkrankungen an nationale oder internationale Kooperationspartner weitergegeben werden, dort für wissenschaftliche Projekte ausgewertet und für zukünftige wissenschaftliche Fragestellungen gelagert werden. Die Proben und Daten werden nur im Rahmen wissenschaftlicher Projekte verwendet und nach spätestens 20 Jahren vernichtet. Durch die Weitergabe der Daten und Proben an wissenschaftliche Kooperationspartner und Forschungsverbünde haben Sie weder eine Belastung noch persönliche Vorteile. Aber durch die Bündelung von Informationen werden sich weitere Themen erforschen lassen, die wir alleine mit der SUPPORT-Studie nicht beantworten können.

Sie haben das Recht, Auskunft über die Sie betreffenden aufgezeichneten Angaben und die Ergebnisse Ihrer Untersuchung bzw. Behandlung zu verlangen, soweit dies nicht aus technischen Gründen niemandem mehr möglich ist. Sollten Sie einer Weiterverarbeitung Ihrer Daten widersprechen, werden keine weiteren Daten über Ihre Person zum Zweck der o. g. Studie erhoben und aufgezeichnet. Sie können bei unrichtiger Aufzeichnung von Angaben, die Ihre Person betreffen, auch eine Berichtigung dieser Angaben verlangen.

Ihre **Ansprechpartner** für Fragen und Informationen im Rahmen der Studie sind:

Dorothea Frevel
Nationales Centrum für Tumorerkrankungen
Tel.: 06221 – 56 35730
E-mail: Dorothea.Frevel@nct-heidelberg.de

Dr. Christine Tjaden
Chirurgische Klinik, Heidelberg
Tel.: 06221 – 56 39694
E-mail: Christine.Tjaden@med.uni-heidelberg.de

Studienleiter:

Prof Dr. Karen Steindorf
Tel.: 06221-42-2351 und -56-5838
Email : karen.steindorf@nct-heidelberg.de

Prof. Dr. Thilo Hackert
Tel.: 06221-56-5950
Email: thilo.hackert@med.uni-heidelberg.de

Dr. Joachim Wiskemann
Tel.: 06221-56-5904
Email: joachim.wiskemann@nct-heidelberg.de

Prof. Dr. Cornelia Ulrich
Tel: 06221-56 5528
Email: neli.ulrich@nct-heidelberg.de

Prof. Dr. Karen Steindorf

Prof. Dr. Thilo Hackert

Dr. Joachim Wiskemann

Prof. Dr. Cornelia Ulrich

Einwilligungserklärung

SUPPORT - Studie

Supervidiertes, **pro**gressives K**r**aft**t**raining für Pankreaskarzinompatienten und -innen: eine randomisierte, kontrollierte Interventionsstudie

Studienteilnehmer/-in: ______________________,________________________

(Name) (Vorname)

Teilnehmer Nr.: _____________ (wird von der Studienzentrale ausgefüllt)

Ich erkläre mich bereit, an der Studie teilzunehmen.

- Die schriftliche Patienten-Information zur Studie habe ich erhalten und gelesen.
- Darüber hinaus bin ich mündlich aufgeklärt worden. Dabei wurden alle meine Fragen beantwortet.
- Im Rahmen der Studie werden keine ärztlichen Diagnosen gestellt.
- Ich bin damit einverstanden, dass ich telefonisch über mitteilungsrelevante Zufallsuntersuchungsergebnisse im Rahmen des Ausdauerleistungstests informiert werde.
- Ich stimme der Teilnahme an der Studie freiwillig zu. Ich weiß, dass ich diese Zustimmung jederzeit ohne Angabe von Gründen und ohne Nachteile für meine weitere medizinische Versorgung widerrufen kann.

Datenschutz

Ich bin mit der Aufzeichnung der im Rahmen der Studie an mir erhobenen Krankheitsdaten und Proben und ihrer pseudonymisierten Verwendung, z. B. für statistische Auswertungen, Veröffentlichungen oder für zukünftige wissenschaftliche Fragestellungen, einverstanden. Ich bin mit der Weitergabe meiner klinischen Daten aus dem Klinikum Ludwigshafen an das Studienzentrum einverstanden. Auch der Weitergabe der pseudonymisierten Daten und Proben an Dritte (Kooperationspartner, Konsortien im In- und Ausland) stimme ich zu. Dritte erhalten keinen Einblick in Originalunterlagen. „Pseudonymisiert" bedeutet, dass ihre Untersuchungsdaten nicht mit Namen oder Adresse gespeichert, sondern mit einer Codenummer versehen und nur in dieser Form wissenschaftlich ausgewertet werden. Die Zuordnung der Daten oder Proben zu einer Person ist nur möglich, wenn hierfür der Schlüssel eingesetzt wird, mit dem die Daten pseudonymisiert wurden.

Eine Kopie der Patienteninformation und der Einwilligungserklärung habe ich erhalten. Das Original verbleibt in der Studienzentrale des Nationalen Centrum für Tumorerkrankungen (NCT).

____

(Datum und Unterschrift des/der Patienten/-in)

Aufklärende Person: ______________________, ______________________

(Name) (Vorname)

____

(Datum und Unterschrift des/der Aufklärenden)

Unterschriften zur Prüfplangenehmigung

Der vorliegende Prüfplan zur SUPPORT-Studie (Version 1.0 vom 30.07.2013) wurde kritisch geprüft und von den unterzeichneten Personen genehmigt. Die Studie wird entsprechend dem vorliegenden Prüfplan und im Einklang mit den Bestimmungen der ICH-GCP (Good Clinical Practice; z.B. GCP Leitlinie CPMP / ICH/135/95) und der Deklaration von Helsinki sowie der GCP-Verordnung (GCP-V) vom 14.08.2004 durchgeführt werden.

Prof Dr. Karen Steindorf

Prof. Dr. Jens Werner

Dr. Joachim Wiskemann

Prof. Dr. Cornelia Ulrich
